# Decreased expression of Calpain-9 predicts unfavorable prognosis in patients with gastric cancer

**DOI:** 10.1038/srep29604

**Published:** 2016-07-12

**Authors:** Peike Peng, Weicheng Wu, Junjie Zhao, Shushu Song, Xuefei Wang, Dongwei Jia, Miaomiao Shao, Mingming Zhang, Lili Li, Lan Wang, Fangfang Duan, Ran Zhao, Caiting Yang, Hao Wu, Jie Zhang, Zhenbin Shen, Yuanyuan Ruan, Jianxin Gu

**Affiliations:** 1 Department of Biochemistry and Molecular Biology, School of Basic Medical Sciences, Fudan University, Shanghai 200032, China; 2 Department of General Surgery, Zhongshan Hospital, Fudan University, Shanghai 200032, China; 3 Institute of Biomedical Science, Fudan University, Shanghai 200032, China

## Abstract

Calpain-8 and calpain-9 belong to the family of calcium-dependent cysteine proteases, which are highly expressed in the stomach. However, the roles of calpain-8 and calpain-9 in gastric tumorigenesis remain little understood. Herein, we demonstrated that calpain-9 was generally decreased in gastric cancer cell lines and primary tumor tissues, while calpain-8 expression was not significantly altered. Calpain-9, but not calpain-8, induced cell cycle arrest in the G1 phase and cellular apoptosis *in vitro*, and it attenuated the growth of subcutaneous tumor xenografts *in vivo*. Low expression of calpain-9 was positively associated with male sex, late T stage, lymph node metastasis, and advanced TNM stage. Further analysis identified calpain-9 as an independent prognostic factor for poor prognosis, and combining calpain-9 with TNM stage generated a better predictive model for patient outcomes. In conclusion, calpain-9 is a tumor suppressor that can be regarded as a potential prognosis indicator for clinical outcomes in gastric cancer.

Gastric cancer is the fifth most common type of cancer and the third leading cause of cancer-related mortality globally, especially in Asia^1^. Although significant improvement has been achieved in surgical techniques and adjuvant treatment, the prognosis of patients with gastric cancer remains poor, with a 5-year survival rate of less than 23%^2^. Gastric cancer is often diagnosed in advanced stages, when available treatments are mostly inefficient^3^. Lack of early detection and effective medical treatment is a crucial reason for the high morbidity and mortality rates of gastric cancer^4^,^5^. Increasing evidence has shown that patients in the same stage might have quite different outcomes due to the heterogeneity of tumors^6^,^7^. Hence, new molecular markers and potential mechanisms are urgently needed and might provide novel therapeutic targets for patients with gastric cancer.

Calpain is a family of intracellular Ca^2+^ -regulated cysteine proteases, evolutionarily well-conserved from bacteria to mammals^8^. The family more than ten members of ubiquitous or tissue-specific proteases that proteolyze a variety of substrates, leading to their degradation or functional modulation^8^. Calpain activity has been implicated in several fundamental physiological processes, including cytoskeletal remodeling, cellular signaling, apoptosis and cell survival^9^. Altered expression of calpain is observed in numerous pathological conditions, including neurodegeneration, myocardial infarction, multiple sclerosis and cancers^9^. Among the members of tissue-specific calpains, calpain-8 is stomach-specific, while calpain-9 is also expressed predominantly in the stomach and small intestine^10^,^11^. CAPN8^−/−^ and CAPN9^−/−^ mice are susceptible to ethanol-induced gastric mucosal injury, indicating that both proteins play protective roles in the gastric mucosa by forming a protease complex^12^. Nevertheless, the roles of calpain-8 and calpain-9 in gastric carcinogenesis remain little understood. In this study, we examined the potential effects of calpain-8 and calpain-9 on tumor growth *in vitro* and *in vivo*, and we also determined the clinical significance of calpain-9 expression as well as its correlation with gastric cancer progression.

## Results

### Calpain-9 expression is decreased in gastric cancer

**Figure 1:**
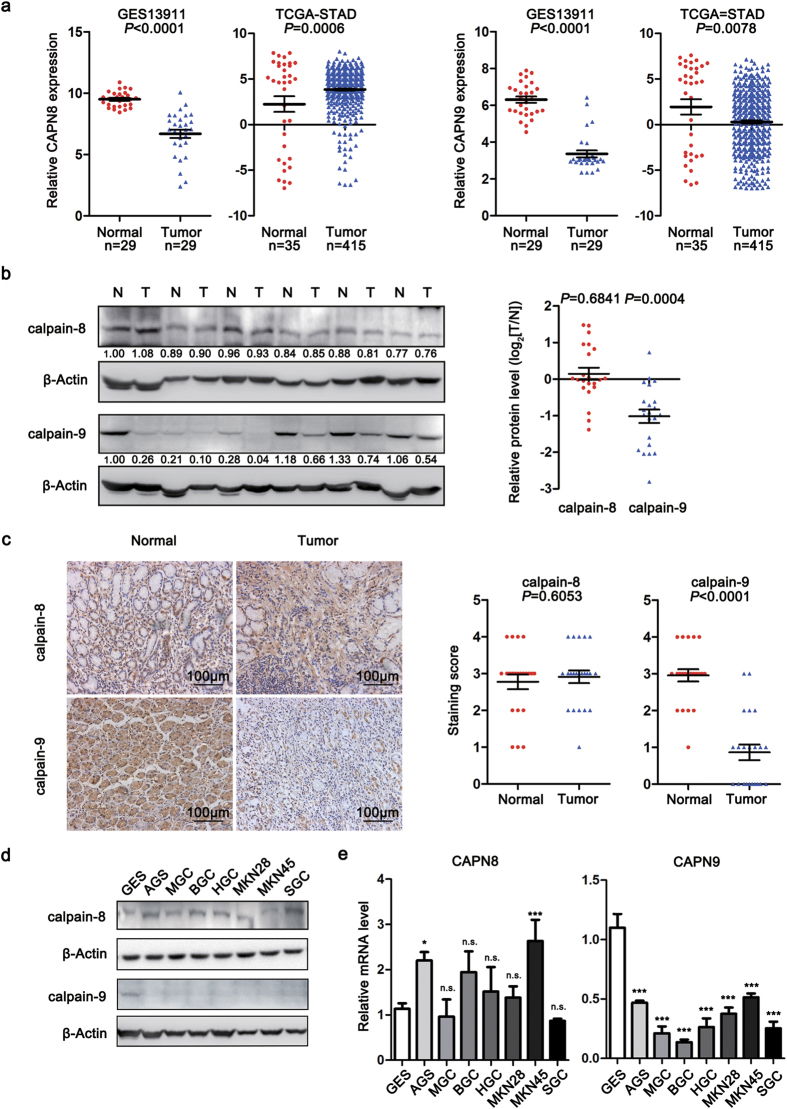
The expression patterns of calpain-8 and calpain-9 in gastric cancer tissues and cell lines. (**a**) Relative expression of CAPN8 and CAPN9 in gastric cancer and normal gastric mucosa tissues in GSE13911 and TCGA-STAD databases. (**b**) The protein levels of calpain-8 and calpain-9 in 22 cases of gastric cancer, and paired adjacent normal tissues were determined by western blot. The images shown are the results of 6 representative cases. N, adjacent normal tissues; T, matched gastric cancer tissues. (**c**) Immunohistochemical analysis of calpain-8 and calpain-9 expression in 22 cases of gastric cancer and paired adjacent normal tissues. The images shown are representative of each group. Scale bar = 100 μm. (**d**,**e**) The protein and mRNA levels of calpain-8 and calpain-9 in GES-1 and seven gastric cancer cell lines were examined by western blot (**d**) and real-time PCR (**e**) analysis. The gels were run under the same experimental conditions. Full-length blots are presented in the Supplement. In (**e**) the statistics were made by comparing with GES-1 group, respectively. *P < 0.05; ***P < 0.001; n.s., no significance.

To understand whether calpain-8 and calpain-9 were involved in gastric carcinogenesis, we first screened differentially expressed genes and examined the mRNA expression patterns of calpain family in gastric cancer tissues from reported GEO^13^ and TCGA-STAD^14^ datasets (Supplementary Fig. S1 and Supplementary Table S1). We found that the calpain-9 mRNA expression was decreased in tumor tissues in both GEO and TCGA datasets, and also displayed the highest fold change among the members of calpain family (Fig. 1a and Supplementary Fig. S1b). Nevertheless, the relative mRNA expression of calpain-8 was reduced in gastric cancer from the GSE13911 dataset, whereas its mRNA levels were increased in gastric cancer from the TCGA-STAD dataset (Fig. 1a and Supplementary Fig. S1b).

We next investigated the protein expression of calpain-8 and calpain-9 in 22 paired gastric cancer samples. Western blot analysis revealed that calpain-9 protein levels were down-regulated in gastric cancer compared with matched adjacent normal gastric mucosa, while calpain-8 protein levels were not significantly altered (Fig. 1b). Immunohistochemical (IHC) assay also confirmed that the protein expression of calpain-9, but not calpain-8, was decreased in gastric cancer samples (Fig. 1c). We also examined the expression of calpain-8 and calpain-9 in normalized gastric mucosa cell line GES-1 and various gastric cancer cell lines (AGS, MGC80-3, BGC-823, HGC-27, MKN-28, MKN-45, and SGC-7901). Results indicated that by comparing with GES-1 cell line, the expression of calpain-9 at the mRNA and protein levels was significantly decreased in all gastric cancer cell lines (Fig. 1d,e). However, the protein levels of calpain-8 in gastric cancer cell lines were comparable with that in GES-1 cells (Fig. 1d). The changes of calpain-8 mRNA levels in different gastric cancer cell lines were also discordant (Fig. 1e). These results suggested that calpain-9 expression is decreased in gastric cancer.

It has been well recognized that chronic *Helicobacter pylori* (*H. pylori*) infection is a risk factor for gastric carcinogenesis. To understand whether calpain-8 or -9 expression in the stomach was affected by *H. pylori*, we next examined their expression patterns in *H. pylori*-infected mice model^15^. However, we found that the mRNA or protein levels of calpain-8 and -9 were not significantly altered in *H. pylori*-infected gastric tissues, compared with normal tissue (Supplementary Fig. S2).

### Calpain-9, but not calpain-8, induces cell cycle arrest at G1 the phase and cellular apoptosis in gastric cancer cells

**Figure 2:**
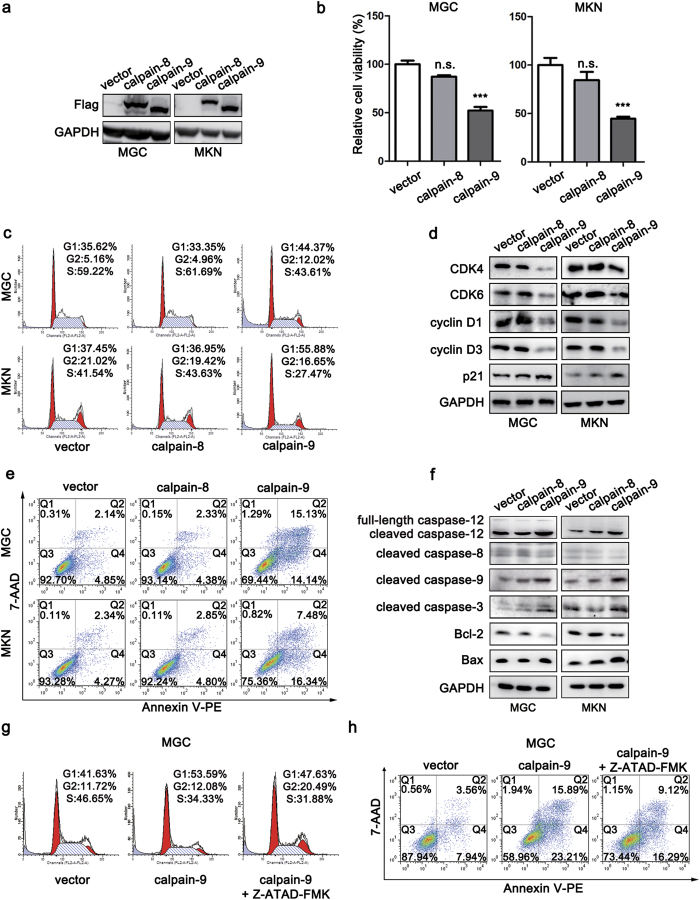
Calpain-9, but not calpain-8, induces cell cycle arrest at the G1 phase and cellular apoptosis in gastric cancer cells. (**a**) The ectopic expression of calpain-8 and calpain-9 in MGC and MKN cells was detected by western blot. (**b**) CCK-8 assays showing the effects of calpain-8 and calpain-9 on cell viability. (**c**,**d**) Flow cytometry (**c**) and western blot (**d**) assays showing the effects of calpain-8 and calpain-9 on cell cycle progression and expression of G1/S transition-related proteins, respectively. (**e**,**f**) Annexin staining (**e**) and western blot (**f**) assays showing the effects of calpain-8 and calpain-9 on cell apoptosis and the expression of apoptosis-related proteins, respectively. (**g**,**h**) The effects of caspase-12 inhibitor Z-ATAD-FMK on cell cycle progression (**g**) and apoptosis (**h**) in calpain-9-transfected MGC80-3 cells. The gels were run under the same experimental conditions. Full-length blots are presented in the Supplement. In (**b**) the statistics were made by comparing with vector group, respectively. ***P < 0.01; n.s., no significance.

To understand better the roles of calpain-8 and calpain-9 in gastric carcinogenesis, stable gastric cancer cell lines (MGC80-3 and MKN-45) that overexpressed calpain-8 or calpain-9 were generated (Fig. 2a). CCK-8 assay revealed that cell viability was reduced in calpain-9-transfected gastric cancer cells, while calpain-8 overexpression had little effect on cell viability (Fig. 2b).

We next evaluated whether calpain-9 modulated cell cycle progression to affect cell viability. PI staining analysis by flow cytometry demonstrated that overexpression of calpain-9 led to a significant increase in the percentage of cells at the G1 phase and a decrease in cells at the S phase (Fig. 2c). We also determined the effects of calpain-9 on the expression of G1/S transition-related cell cycle proteins. Western blot analysis showed that overexpression of calpain-9 suppressed the expression of key regulators in the G1 phase, including cyclin D1, cyclin D3 and CDK4/6, and it elevated the levels of cyclin-dependent kinase inhibiter p21 (Fig. 2d). However, overexpression of calpain-8 showed little influence on cell cycle progression or the expression of cell cycle-related proteins (Fig. 2c,d). These results suggested that calpain-9, but not calpain-8, induces cell cycle arrest at the G1 phase in gastric cancer cells.

We next determined the effects of calpain-8 and calpain-9 on cellular apoptosis in gastric cancer cells. Annexin V staining revealed that overexpression of calpain-9, but not calpain-8, significantly increased cell apoptosis in both gastric cancer cell lines (Fig. 2e). Western blot analysis also demonstrated that transfection of calpain-9 increased the levels of cleaved caspase-9 and cleaved caspase-3, but it attenuated the expression of Bcl-2 in both cell lines (Fig. 2f). In addition, overexpression of calpain-9 also up-regulated the protein level of Bax in MKN-45 cells (Fig. 2f). These results suggested that calpain-9 induces cell apoptosis in gastric cancer cells.

Previous studies have suggested that ubiquitously expressed calpain-1 and -2 proteases might promote apoptosis through a calpain-caspase-12-caspase-3 cascade^16^. Because calpain-8 and -9 share high structural similarity with calpain-1 and -2, we next assessed whether calpain-8 or calpain-9 influenced the activation of caspase-12. Western blot analysis revealed that transfection with calpain-9, but not calpain-8, enhanced the cleavage of caspase-12 in both gastric cancer cell lines (Fig. 2f). We also determined whether activation of caspase-12 was involved in the anti-tumorigenic effect of calpain-9 using the specific caspase-12 inhibitor Z-ATAD-FMK. Cell cycle analysis showed that G1 phase arrest in calpain-9-overexpressed gastric cancer cells was blocked in the presence of Z-ATAD-FMK (Fig. 2g). In addition, inhibition of caspase-12 also suppressed calpain-9-induced apoptosis in MGC80-3 cells (Fig. 2h). Therefore, activation of caspase-12 might contribute to both G1/S phase block and apoptosis mediated by calpain-9 in gastric cancer.

### Calpain-9 attenuates subcutaneous tumor growth in nude mice

**Figure 3:**
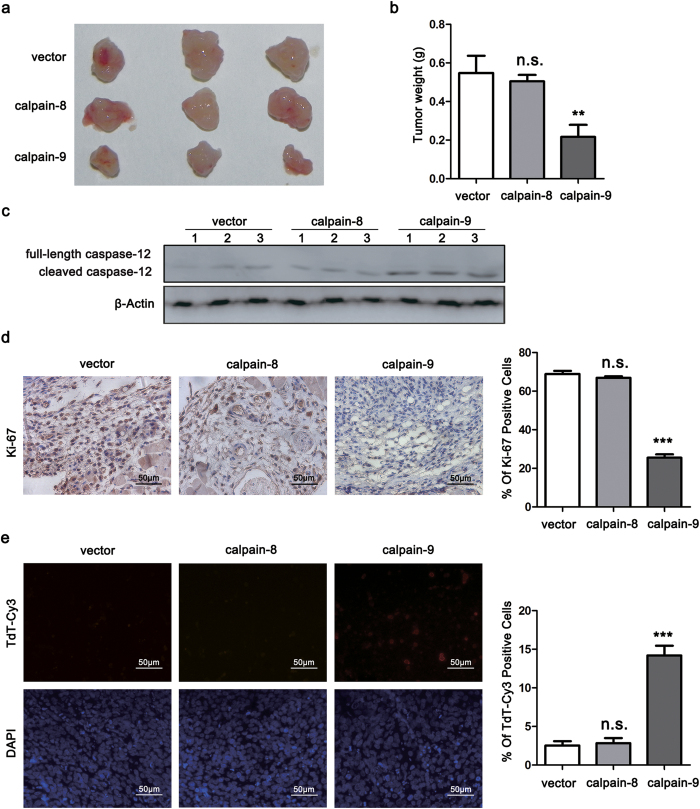
Calpain-9, but not calpain-8, attenuates subcutaneous tumor growth in nude mice. Subcutaneous tumor models were established described in the “Materials and Methods” section. After sacrifice of the mice, tumor xenografts were collected. (**a**) Representative images of tumors in three groups. (**b**) Tumor weight in each group was evaluated. (**c**) The cleavage of caspase-12 in tumor tissues was determined by western blot. (**d**,**e**) Immunohistochemistry staining was performed to detect Ki-67 expression (**d**) and TUNEL assay was performed to detect cellular apoptosis (**e**). The gels were run under the same experimental conditions. Full-length blots are presented in the Supplement. Scale bar, 50 μm. In (**b**,**d**,**e**) the statistics were made by comparing with vector group, respectively. **P < 0.01; ***P < 0.001; n.s., no significance.

To identify further the effects of calpain-8 and calpain-9 on gastric tumor growth *in vivo*, subcutaneous tumor model was established with stable MGC80-3 cells. We found that tumor size and weight were significantly suppressed in the calpain-9 group, compared with those in the calpain-8 group and control group (Fig. 3a,b). Western blot analysis also confirmed that the calpain-9 group displayed higher protein levels of cleaved caspase-12 than the calpain-8 and control groups (Fig. 3c). Moreover, overexpression of calpain-9 also induced a remarkable reduction in the expression of the proliferation marker Ki-67 and significantly increased cellular apoptosis by TUNEL staining (Fig. 3d,e). These results suggested that calpain-9, but not calpain-8, attenuates the growth of gastric cancer *in vivo*.

### Correlations between calpain-9 expression and clinicopathological features in gastric cancer patients

**Figure 4:**
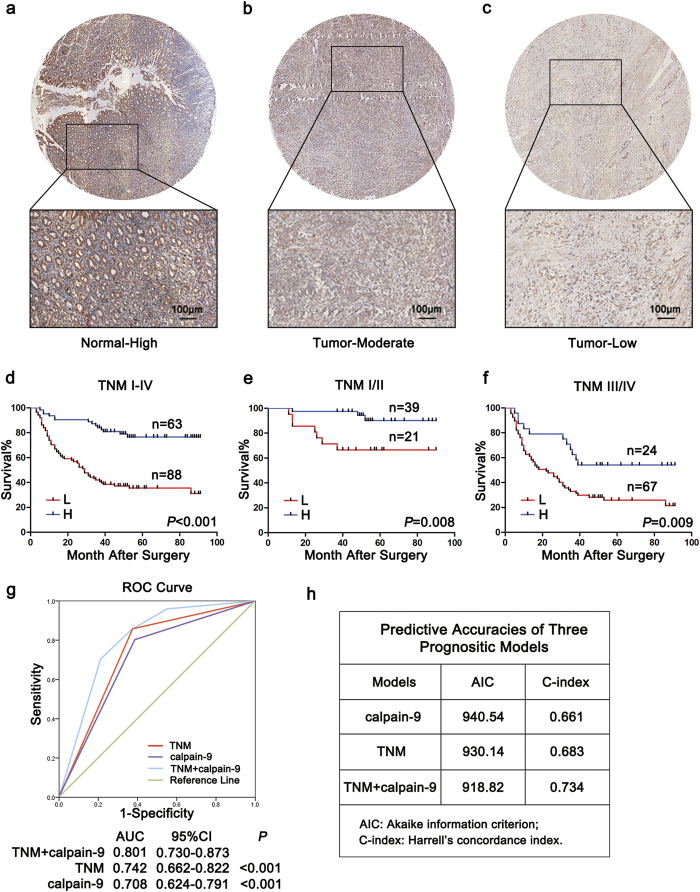
The predictive value of calpain-9 expression in patients with gastric cancer. (**a**–**c**) Representative images of tissue microarray stained for calpain-9 and its regional magnification in gastric cancer sections and adjacent normal sections. Adjacent normal sections showed high calpain-9 expression (**a**) while gastric cancer tissues showed moderate to low calpain-9 expression (**b**,**c**). Bar: 100 μm. (**d–f**) Kaplan-Meier survival analysis showing the relationship between calpain-9 expression and overall survival in all patients (**d**) patients at TNM I-II stage (**e**) and patients at TNM III-IV stage (**f**). (**g**) Receiver operating characteristic curve analysis of the sensitivity and specificity of the predictive value of the TNM stage model, calpain-9 model and combined TNM stage and calpain-9 stratification model. (**h**) The Akaike information criterion (AIC) and Harrell’s concordance index (C-index) analysis of the comparison of the predictive accuracies of TNM staging and calpain-9 expression.

Because calpain-9, but not calpain-8, was decreased in gastric cancer and displayed anti-tumorigenic effects *in vitro* and *in vivo*, we next determined the correlations between calpain-9 expression and clinicopathological features in 151 gastric cancer samples. Calpain-9 expression was apparently lower in gastric cancer tissues than in non-tumor gastric mucosa (Fig. 4a–c). The association between calpain-9 expression and clinicopathological variables in gastric cancer patients were analyzed by the chi-square test, and they are listed in Table 1. Among the variables, low expression of calpain-9 was positively associated with male sex, late T stage, lymph node metastasis, and advanced TNM stage. These data suggested that low intratumoral calpain-9 expression is correlated with gastric cancer progression.

### Correlations between calpain-9 expression and overall survival in gastric cancer patients

We next explored the relationship between calpain-9 expression and overall survival using Kaplan-Meier analysis. The results demonstrated that high expression of calpain-9 in tumor tissues showed a survival benefit in gastric cancer patients (Fig. 4d). To evaluate further the efficiency of calpain-9 expression in stratifying patients with different TNM stages, we divided the patients into early (I-II) and advanced (III-IV) groups, respectively. In both the TNM I+II and TNM III+IV subgroups, calpain-9 expression showed statistically significant value in predicting the outcomes of gastric cancer patients (Fig. 4e,f). These data suggested that calpain-9 expression is correlated with overall survival for patients with gastric cancer.

We also conducted univariate Cox analysis to identify the prognostic significance of clinicopathological factors for overall survival. Vessel invasion (*P* < 0.001), T stage (*P* < 0.001), lymph node metastasis (*P* < 0.001), distant metastasis (*P* = 0.046), TNM stage (*P* < 0.001), and calpain-9 expression (*P* < 0.001) were found to be risk factors for survival in patients with gastric cancer (Table 2). Further adjustment of covariate factors using multivariate Cox analysis identified T stage (*P* = 0.031), lymph node metastasis (*P* = 0.023) and calpain-9 expression (*P* = 0.003) as independent risk factors for gastric cancer (Table 2). These data indicate that low expression of calpain-9 is an independent factor that predicts poor prognosis in patients with gastric cancer.

### Combination of calpain-9 expression with TNM stage generates a better predictive model for overall survival of gastric cancer patients

To establish a more sensitive model for predicting the outcomes of patients with gastric cancer, we combined calpain-9 expression and TNM stage to create a prognostic score system. ROC (receiver operating characteristic) curve analysis showed that the combination of calpain-9 and TNM stage revealed better prognostic value (AUC [95% CI], 0.801 [0.730–0.873]) than calpain-9 expression alone (AUC [95% CI], 0.708 [0.624–0.791]) or TNM stage alone (AUC [95% CI], 0.742 [0.662–0.822]) with statistical significance (Fig. 4g). In addition, the AIC was 930.14 when estimated according to TNM stage alone, and it decreased to 918.82 when estimated in combination with calpain-9 expression (Fig. 4h). The C-index was 0.683 when estimated according to TNM stage alone, and it increased to 0.734 when calpain-9 expression was added (Fig. 4h).

We also constructed a nomogram model that integrated TNM classification with calpain-9 expression for better stratifying patients with different prognosis. In this nomogram, a higher total point predicts a worse prognosis. The total point was raised by adding the score of the TNM classification (0 for “I”, 33 for “II”, 67 for “IIIA” or 100 for “IV”) and calpain-9 expression (29 for “Low” or 0 for “High”) for each patient (Supplementary Fig. S3a). The calibration curve for predicting 5-year overall survival showed that the nomogram performed well with the ideal prediction model (Supplementary Fig. S3b). In addition, the Harrell’s c-index for the generated nomogram was 0.738, higher than that for TNM stage (0.696), indicating that the nomogram generated gave a better prediction for overall survival of the patients than applying TNM staging system alone (Supplementary Fig. S3c). These results suggest that incorporation of calpain-9 expression into TNM stage could establish a better predictive model for the overall survival of gastric cancer patients.

## Discussion

As with other cancers, gastric cancer is a highly heterogeneous disease with poor clinical outcomes. In addition to traditional tumor-node-metastasis (TNM) stage, gastric cancer is divided into intestinal, mixture and diffuse types by the Lauren classification system^17^,^18^. These classification systems provide a predictive model for patients, but they still have limited capacity to determine different outcomes when referring to the molecular heterogeneity of gastric cancer^7^. Therefore, more molecular mechanism studies should be undertaken to understand the progression of gastric cancer. Consequently, our results demonstrated that expression of calpain-9 was decreased in gastric cancer and was associated with the prognosis of patients in gastric cancer. In addition, calpain-9 could create a better predictive model for the outcomes of gastric cancer patients in the combination with TNM stage. It might provide new therapeutic approaches for gastric cancer by targeting calpain-9 as a clinically useful prognostic marker.

Calpains constitute a superfamily of intracellular Ca^2+^-activated non-lysosomal cysteine proteases, and they are divided into typical (1, 2, 3, 8, 9, 11, 12, and 14) and atypical calpains (5, 6, 7, 10, 13, and 15), as well as according to whether the protease has a penta-EF hand in domain IV^19^. Increased expression of calpains is generally observed in several types of cancers, and might play oncogenic roles in promoting the malignancy of cancers^8^. Ubiquitous calpains (1 and 2) are highly expressed in colorectal cancer and prostate cancer^20^,^21^. Expression of atypical Caplain-6 is increased in osteosarcoma cells and uterine cancer cells, contributing to the facilitation of cell migration and proliferation^22^,^23^. However, unlike other members of the calpain family, we found that calpain-9 was down-regulated in gastric cancer in our study. In addition, our results also indicated that the protein expression of stomach-specific calpain-8 was not altered in gastric cancer and showed little effect on tumor growth *in vitro* and *in vivo*, while the opposite changes were reported in the mRNA levels of calpain-8 between two previous GEO and TCGA datasets. Therefore, the role of calpain-8 in gastric cancer needs to be clarified in further research.

Among the members of tissue-specific calpains, calpain-8 is stomach-specific, while calpain-9 is expressed predominantly in the stomach and small intestine^10^,^11^. However, the roles of calpain-8 and -9 during various physiological and pathophysiological conditions are not well understood. A recent study demonstrated that calpain-8 and calpain-9 evolved to have local structures specific for binding to each other and forming a protease complex to play a protective role in gastric mucosal defense^12^. In that report, the presence of calpain-9 alone or inactive calpain-8 with its proteolyzed fragments was insufficient for the effective protection mechanism, suggesting that proteolytic activity of calpain-8 is essential for gastric mucosal defense. Nevertheless, in transformed gastric mucosa, calpain-9, but not calpain-8, might function as a tumor suppressor through proliferation suppression and apoptosis induction. The differential role of calpain-8 and -9 in gastric tumorigenesis occurs possibly due to the different substrates that they proteolyze. Although calpain-8 and -9 have a typical domain structure like that of calpain-1 and -2, calpain-9 must bind to the calpain regulatory subunit (calpain-4) for activation while calpain-8 does not^24^,^25^. Therefore, association with calpain-4 might help in the determination of substrate specificity. Furthermore, crystal structure studies have indicated that the functions of the calpain-9 domains dIII and dIV differ from their function in the ubiquitous calpains, suggesting that calpain-9 might act on different substrates and might exhibit diverse mechanisms of action^26^.

Nowadays, gastric cancer remains the fifth most common cancer and the third leading cause of cancer-related mortality worldwide^1^. While earlier diagnosis has led to prolonged survival, patients with advanced gastric cancer still have poor clinical outcomes^27^. Although the utility of classic chemotherapy agents has been explored, advances have been slow and the efficacy of these agents has reached a plateau. Therefore, targeted therapies, whether as single-agent therapy or in combination with traditional therapies, could have potential impact on the improvement of the overall prognosis of gastric cancer. Our data suggested that calpain-9 could be useful as a new biomarker to establish the risk and prognosis of gastric cancer and to facilitate the selection of therapeutic modalities in clinical practice, as well as to propose a strategy to target calpain-9 as a potential adjuvant therapy for gastric cancer treatment.

## Methods

### Primary gastric cancer samples

All of the methods were approved by the research medical ethics committee of Fudan University and were performed in accordance with the approved guidelines. Primary tumor specimens of gastric cancer were obtained from 151 gastric cancer patients who underwent gastrectomy without preoperative treatment in the Department of General Surgery, Zhongshan Hospital (Fudan University, Shanghai, China), between 2004 and 2008. Relevant clinicopathological features and survival data were extracted from hospital records. Frozen gastric cancer and matched adjacent normal mucosa tissues were also obtained from the Department of General Surgery, Zhongshan Hospital (Fudan University, Shanghai, China) in 2014. Adjacent normal mucosa tissues were obtained from sites that were >60 mm away from primary lesions. The study was approved by the Research Ethics Committee of Zhongshan Hospital, and informed consent was obtained from every patient.

### TCGA and GEO datasets

These data are publically available from the Cancer Genome Atlas and the NCBI Gene Expression Omnibus (accession number: GSE13911). For the TCGA dataset, all level-3 data were downloaded from the TCGA-STAD portal by using TCGA-Assembler software^14^. The mRNA expression in TCGA dataset was measured by RNA sequencing V2. The RSEM (RNA-Seq by Expectation-Maximization) counts were further normalized by TMM (trimmed mean of M value) method to estimate the relative RNA production levels using edgeR software^28^. Then we performed voom analysis to estimate the mean-variance relationship of the log-count^29^. For the GSE13911 dataset reported previously, the mRNA expression was measured by microarray. The probe set intensities were quantified using the GeneChip Operating Software (GCOS) and normalized with GCRMA (GeneChip Robust Multiarray Averaging) using Array Assist Software. Differentially expressed genes in TCGA and GSE13911 datasets were screened with the paired moderated t-test using limma software package^30^, and described in Supplementary Fig. 1a and Supplementary Table S1.

### Cell lines

Seven gastric cancer cell lines (AGS, MGC80-3, BGC-823, HGC-27, MKN-28, MKN-45, SGC-7901) and a gastric mucosal cell line (GES-1) were obtained from the Cell Bank of Type Culture Collection of Chinese Academy of Sciences (Shanghai, China). HGC27, MKN-28, and MKN-45 cells were cultured in Dulbecco’s Minimum Essential Medium and the other cell lines were cultured in RPMI-1640 Medium, supplemented with 10% fetal bovine serum at 37 °C in a humidified atmosphere containing 5% CO_2_. All of the culture media were purchased from Sigma (St. Louis, MO, USA), and fetal bovine serum was purchased from Gibco (catalogue no. 16000-044; Grand Island, NY, USA).

### Western blotting

Protein from cell lysates or tissues lysates were separated by SDS–polyacrylamide gel electrophoresis, transferred onto polyvinylidene difluoride membranes, and incubated with primary antibodies, including: calpain-9 (1:1000; Proteintech, USA), calpain-8 (1:1000; Abcam, USA), cyclin D1 (1:500; Cell Signaling, USA), cyclin D3 (1:1000; Cell Signaling), CDK4 (1:1000; Cell Signaling), CDK6 (1:1000; Cell Signaling), p21 (1:1000; Cell Signaling), cleaved caspase-3 (1:500; Cell Signaling), cleaved caspase-8 (1:1000; Cell Signaling), cleaved caspase-9 (1:1000; Cell Signaling), and caspase-12 (1:1000; Proteintech), followed by incubation with horseradish peroxidase (HRP)-conjugated secondary antibody (1:2000; Santa). Protein expression was visualized by enhanced chemiluminescence assay.

### Quantitative real-time PCR

Total RNA was purified from cancer cells using TRIzol (Invitrogen, Carlsbad, CA, USA) according to the manufacturer’s instructions. RNA was used for the first-strand cDNA synthesis with a Takara RNA PCR Kit (Takara, Dalian, China), and the resulting DNA was analyzed by quantitative real-time PCR using SYBR Premix Ex Taq (Takara) according to the manufacturer’s instructions. GAPDH was used as an internal control. The primers used were as follows: CAPN8 forward, 5′-TGGCTCCAACCAAAACGCTT-3′; CAPN8 reverse, 5′-CCTGGTCCAAGATCCTAGC-3′; CAPN9 forward, 5′-AGAGGCACCCTG TTTGAGGAT-3′; CAPN9 reverse, 5′-CCCCTGGTCGTTTCCACAC-3′; GAPDH, forward, 5′-AAGGTCGGAGTCAACGGATTTG-3′; and GAPDH reverse, 5′-CCATGGGTGGAATCATATTGGAA-3′.

### Immunohistochemistry

Tissue microarray on glass slides was deparaffinized in xylene, dehydrated in graded ethanol and subjected to antigen retrieval in boiling citrate buffer (0.01 M, pH 6.0). Then, the sections were blocked by UltraVision Hydrogen Peroxide Block (Thermo Scientific, CA, USA) and UltraVision Protein Block (Thermo Scientific), followed by primary antibody incubation. UltraVision Quanto Detection System horseradish peroxidase (HRP) Polymer (Thermo Scientific) and DAB Quanto (Thermo Scientific) were applied staining and hematoxylin was used for counterstaining. Immunohistochemical scoring was determined according to our previous report^31^.

### Plasmids construction, transfection and stable cell lines

The cDNA encoding CAPN8 and CAPN9 was obtained by PCR and was inserted into the pCMV-Flag vector (Sigma, St. Louis, MO, USA). Transfections were performed with Lipofectamine 3000 (Life Technologies, CA, USA), according to the manufacturer’s instructions. Stable cell lines were selected with G418 (200 μg/mL) in the medium.

### Cell viability assay

Cell viability was quantified with a Cell Counting Kit-8 (CCK-8) (Dojindo, Japan), according to the manufacturer’s instructions. The cells were plated at a density of 3,000 cells per well in 96-well plates. The CCK-8 assays were assessed at 24 h after transfection by measuring the absorbance at 450 nm.

### Cell cycle and apoptosis assay

Cycle arrest and apoptotic cells were detected by flow cytometric analysis. After transfection for 36 h, cells were collected by trypsinization and washed twice with PBS. For cell cycle assay, the collected cells were stained with propidium iodide (PI) using a Cell Cycle Staining Kit (Lianke Bio, Hangzhou, China). Cellular apoptosis was determined with annexin V-PE and 7-AAD using PE Annexin V Apoptosis Detection Kit I (BD Biosciences, CA, USA). Stained cells were assessed by flow cytometry, and the data were analyzed by FlowJo software (TreeStar, Ashland, OR, USA).

### Subcutaneous xenograft models

All of the animal experiments were approved by the research medical ethics committee of Fudan University and were performed in accordance with the approved guidelines. The nude mice were purchased from Shanghai Laboratory Animal Center of Chinese Academy Sciences and were housed in individual ventilated cages. All of the mice were randomly grouped (n = 6 in each group). MGC80-3 cells (1 × 10^7^ cells in 100 μL PBS) stably transfected with CAPN8 or CAPN9 expression vector were injected subcutaneously into the right hind flank regions of 4-week-old male Balb/c nude mice. The mice were sacrificed after 3 weeks, and tumor tissues were harvested.

### TUNEL assay

Terminal deoxynucleotidyl transferase dUTP nick end labeling (TUNEL) staining was performed to assess apoptosis of subcutaneous tumors sections using a TUNEL apoptosis detection kit (Biyotime Biotech, Jiangsu, China), according to the manufacturer’s instructions. After labeling, the sections were incubated with DAPI (Biyotime Biotech) for 3 min to stain the nucleus. TUNEL positive cells were detected by fluorescence microscopy and were counted.

### Statistical analysis

Analysis was performed with SPSS software, version 22.0 (SPSS, Chicago, IL, USA), and R software (http://www.r-project.org/). The data are presented as the mean ± standard deviation, with at least three replicates used for each data point. Pearson’s chi-square test was used to compare the expression level of calpain-9 and clinical variables. Survival was evaluated by Kaplan-Meier survival curves, and the log-rank test was used to evaluate the differences between groups. Univariate and multivariate survival analyses were performed using Cox regression. Nomogram was constructed using R software, version 3.0.2, with the “rms” package. A calibration plot was generated to examine the performance characteristics of the constructed nomogram. The predictive value of the parameters was assessed by ROC curve analyses, the Akaike information criterion (AIC) and Harrell’s concordance index (C-index). Differences between two groups were analyzed with Student’s t test. Multiple comparisons were performed by one-way ANOVA test followed by dunnett’s post-hoc analysis (if the ANOVA turned out to be significant). Statistical significance was determined at the level of *P* < 0.05.

## Additional Information

**How to cite this article**: Peng, P. *et al*. Decreased expression of Calpain-9 predicts unfavorable prognosis in patients with gastric cancer. *Sci. Rep.* **6**, 29604; doi: 10.1038/srep29604 (2016).

## Supplementary Material

Supplementary Information

## Figures and Tables

**Table 1:** Correlation between calpain-9 expression and clinicopathological features of gastric cancer patients.

Variables	Calpain-9 expression			
	No.	Low	High	*P*-value*
		No. (%)	No. (%)	
Gender				
Male	100	67 (67.0)	33(33.0)	**0.002**
Female	51	21(41.2)	30(58.8)	
Age				
<60	71	37(52.1)	34(47.9)	0.148
>=60	80	51(63.8)	29(36.2)	
Tumor site				
Cardia	23	12(52.2)	11(47.8)	0.514
Body	38	25(65.8)	13(34.2)	
Antrum	90	51(56.7)	39(43.3)	
Lauren type				
Intestinal	96	53(55.2)	43(44.8)	0.371
Mixture	20	11(55.0)	9(45.0)	
Diffuse	35	24(68.6)	11(31.4)	
Differentiation				
Well/Moderately	30	17(56.7)	13(43.3)	0.842
Poorly	121	71(58.7)	50(41.3)	
Vessel invasion				
Positive	50	31(62.0)	19(38.0)	0.514
Negative	101	57(56.4)	44(43.6)	
T stage				
T1–T2	36	10(27.8)	26(72.2)	**<0.001**
T3–T4	115	78(67.8)	37(32.2)	
Lymph node metastasis				
Positive	92	65(70.7)	27(29.3)	**<0.001**
Negative	59	23(39.0)	36(61.0)	
Distant metastasis				
M1	2	2(100.0)	0(0.0)	0.510
M0	149	86(57.7)	63(42.3)	
TNM stage				
I	21	5(23.8)	16(76.2)	**<0.001**
II	39	16(41.0)	23(59.0)	
III	89	65(73.0)	24(27.0)	
IV	2	2(100.0)	0(0.0)	

*P* < 0.05 indicates that the differences have statistical significance.

^*^Pearson chi-square tests.

**Table 2:** Univariate and multivariate Cox regression analysis for overall survival of gastric cancer patients.

Variables	Univariate			Multivariate		
	HR	95% CI	*P*-value	HR	95% CI	*P*-value
Gender						
Male vs female	1.292	0.791–2.110	0.313			
Age (years)						
≥60 vs <60	1.373	0.859–2.195	0.237			
Tumor site						
Cardia+body vs antrum	1.263	0.777–2.053	0.318			
Lauren type						
Diffuse+mixture vs intestinal	1.494	0.920–2.426	0.123			
Differentiation						
Poorly vs well/moderately	1.140	0.638–2.039	0.708			
Vessel invasion						
Positive vs negative	2.711	1.588–4.628	**<0.001**	1.543	0.939–2.534	0.087
T stage						
T3–T4 vs T1–T2	3.516	2.104–5.877	**<0.001**	4.863	1.192–19.847	**0.027**
Lymph node metastasis						
Positive vs negative	4.038	2.516–6.481	**<0.001**	3.425	1.108–10.589	**0.033**
Distant metastasis						
M1 vs M0	19.81	1.234–318.1	**0.046**	1.334	0.314–5.662	0.696
TNM stage						
III–IV vs I–II	4.258	2.656–6.825	**<0.001**	0.867	0.239–3.142	0.828
calpain-9 expression						
High vs low	0.280	0.175–0.450	**<0.001**	0.387	0.209–0.715	**0.002**

CI, Confidence interval; HR, Hazard ratio; *P* < 0.05 was considered to be statistically significant.

## References

[b1] Torre L. A. . Global cancer statistics, 2012. CA: a cancer journal for clinicians 65, 87–108, doi: 10.3322/caac.21262 (2015). 25651787

[b2] Chua Y. J. & Cunningham D. The UK NCRI MAGIC trial of perioperative chemotherapy in resectable gastric cancer: implications for clinical practice. Annals of surgical oncology 14, 2687–2690, doi: 10.1245/s10434-007-9423-7 (2007). 17653804

[b3] Shah M. A. & Kelsen D. P. Gastric cancer: a primer on the epidemiology and biology of the disease and an overview of the medical management of advanced disease. Journal of the National Comprehensive Cancer Network: JNCCN 8, 437–447 (2010). 20410336 10.6004/jnccn.2010.0033

[b4] Liang H. & Kim Y. H. Identifying molecular drivers of gastric cancer through next-generation sequencing. Cancer letters 340, 241–246, doi: 10.1016/j.canlet.2012.11.029 (2013). 23178814 PMC3873853

[b5] Gonzalez C. A. & Agudo A. Carcinogenesis, prevention and early detection of gastric cancer: where we are and where we should go. International journal of cancer. Journal international du cancer 130, 745–753, doi: 10.1002/ijc.26430 (2012). 21918974

[b6] Lim L., Michael M., Mann G. B. & Leong T. Adjuvant therapy in gastric cancer. Journal of clinical oncology: official journal of the American Society of Clinical Oncology 23, 6220–6232, doi: 10.1200/JCO.2005.11.593 (2005). 16135489

[b7] Stock M. & Otto F. Gene deregulation in gastric cancer. Gene 360, 1–19, doi: 10.1016/j.gene.2005.06.026 (2005). 16154715

[b8] Moretti D., Del Bello B., Allavena G. & Maellaro E. Calpains and cancer: friends or enemies? Archives of biochemistry and biophysics 564, 26–36, doi: 10.1016/j.abb.2014.09.018 (2014). 25305531

[b9] Storr S. J., Carragher N. O., Frame M. C., Parr T. & Martin S. G. The calpain system and cancer. Nature reviews. Cancer 11, 364–374, doi: 10.1038/nrc3050 (2011). 21508973

[b10] Sorimachi H., Ishiura S. & Suzuki K. A novel tissue-specific calpain species expressed predominantly in the stomach comprises two alternative splicing products with and without Ca(2+)-binding domain. The Journal of biological chemistry 268, 19476–19482 (1993). 7690035

[b11] Lee H. J., Sorimachi H., Jeong S. Y., Ishiura S. & Suzuki K. Molecular cloning and characterization of a novel tissue-specific calpain predominantly expressed in the digestive tract. Biological chemistry 379, 175–183 (1998). 9524069 10.1515/bchm.1998.379.2.175

[b12] Hata S. . Calpain 8/nCL-2 and calpain 9/nCL-4 constitute an active protease complex, G-calpain, involved in gastric mucosal defense. PLoS genetics 6, e1001040, doi: 10.1371/journal.pgen.1001040 (2010). 20686710 PMC2912385

[b13] D’Errico M. . Genome-wide expression profile of sporadic gastric cancers with microsatellite instability. European journal of cancer 45, 461–469, doi: 10.1016/j.ejca.2008.10.032 (2009). 19081245

[b14] Zhu Y., Qiu P. & Ji Y. TCGA-assembler: open-source software for retrieving and processing TCGA data. Nature methods 11, 599–600, doi: 10.1038/nmeth.2956 (2014). 24874569 PMC4387197

[b15] Carbo A. . Systems modeling of the role of interleukin-21 in the maintenance of effector CD4+ T cell responses during chronic Helicobacter pylori infection. mBio 5, e01243–01214, doi: 10.1128/mBio.01243-14 (2014). 25053783 PMC4120195

[b16] Kerbiriou M., Teng L., Benz N., Trouve P. & Ferec C. The calpain, caspase 12, caspase 3 cascade leading to apoptosis is altered in F508del-CFTR expressing cells. PloS one 4, e8436, doi: 10.1371/journal.pone.0008436 (2009). 20041182 PMC2793515

[b17] Edge S. B. & Compton C. C. The American Joint Committee on Cancer: the 7th edition of the AJCC cancer staging manual and the future of TNM. Annals of surgical oncology 17, 1471–1474, doi: 10.1245/s10434-010-0985-4 (2010). 20180029

[b18] Turner E. S. & Turner J. R. Expanding the Lauren classification: a new gastric cancer subtype? Gastroenterology 145, 505–508, doi: 10.1053/j.gastro.2013.07.019 (2013). 23891604

[b19] Suzuki K., Hata S., Kawabata Y. & Sorimachi H. Structure, activation, and biology of calpain. Diabetes 53 Suppl 1, S12–18 (2004). 14749260 10.2337/diabetes.53.2007.s12

[b20] Rios-Doria J., Kuefer R., Ethier S. P. & Day M. L. Cleavage of beta-catenin by calpain in prostate and mammary tumor cells. Cancer research 64, 7237–7240, doi: 10.1158/0008-5472.CAN-04-1048 (2004). 15492240

[b21] Liu T., Mendes D. E. & Berkman C. E. Prolonged androgen deprivation leads to overexpression of calpain 2: implications for prostate cancer progression. International journal of oncology 44, 467–472, doi: 10.3892/ijo.2013.2196 (2014). 24297527 PMC3898865

[b22] Marion A. . Calpain-6 is an endothelin-1 signaling dependent protective factor in chemoresistant osteosarcoma. International journal of cancer. Journal international du cancer 130, 2514–2525, doi: 10.1002/ijc.26246 (2012). 21681744

[b23] Skubitz K. M. & Skubitz A. P. Differential gene expression in uterine leiomyoma. The Journal of laboratory and clinical medicine 141, 297–308, doi: 10.1016/S0022-2143(03)00007-6 (2003). 12761473

[b24] Lee H. J. . Characterization of a human digestive tract-specific calpain, nCL-4, expressed in the baculovirus system. Archives of biochemistry and biophysics 362, 22–31, doi: 10.1006/abbi.1998.1021 (1999). 9917325

[b25] Hata S., Doi N., Kitamura F. & Sorimachi H. Stomach-specific calpain, nCL-2/calpain 8, is active without calpain regulatory subunit and oligomerizes through C2-like domains. The Journal of biological chemistry 282, 27847–27856, doi: 10.1074/jbc.M703168200 (2007). 17646163

[b26] Davis T. L. . The crystal structures of human calpains 1 and 9 imply diverse mechanisms of action and auto-inhibition. Journal of molecular biology 366, 216–229, doi: 10.1016/j.jmb.2006.11.037 (2007). 17157313

[b27] Lordick F. . Unmet needs and challenges in gastric cancer: the way forward. Cancer treatment reviews 40, 692–700, doi: 10.1016/j.ctrv.2014.03.002 (2014). 24656602

[b28] Robinson M. D. & Oshlack A. A scaling normalization method for differential expression analysis of RNA-seq data. Genome biology 11, R25, doi: 10.1186/gb-2010-11-3-r25 (2010). 20196867 PMC2864565

[b29] Law C. W., Chen Y., Shi W. & Smyth G. K. Voom: Precision weights unlock linear model analysis tools for RNA-seq read counts. Genome biology 15, R29, doi: 10.1186/gb-2014-15-2-r29 (2014). 24485249 PMC4053721

[b30] Ritchie M. E. . limma powers differential expression analyses for RNA-sequencing and microarray studies. Nucleic acids research 43, e47, doi: 10.1093/nar/gkv007 (2015). 25605792 PMC4402510

[b31] Chen L. . Loss of RACK1 Promotes Metastasis of Gastric Cancer by Inducing a miR-302c/IL8 Signaling Loop. Cancer research 75, 3832–3841, doi: 10.1158/0008-5472.CAN-14-3690 (2015). 26199092

