# Supplementary material for: Decreased expression of Calpain-9 predicts unfavorable prognosis in patients with gastric cancer
Source: Sci Rep. 2016 Jul 12;6:29604. doi: 10.1038/srep29604 (PMC4941732; doi:10.1038/srep29604)
Supplement: Supplementary Information [file srep29604-s1.pdf]

Decreased expression of Calpain-9 predicts unfavorable prognosis in patients with gastric cancer

Peike Peng[1], Weicheng Wu[1], Junjie Zhao[2] , Shushu Song[1], Xuefei Wang[2], Dongwei Jia[1], Miaomiao Shao[1], Mingming Zhang[1], Lili Li[1], Lan Wang[1], Fangfang Duan[3], Ran Zhao[3], Caiting Yang[1], Hao Wu[1], Jie Zhang[3], Zhenbin Shen[2,*], Yuanyuan Ruan[1,*], Jianxin Gu[1,3]

Supplementary Data

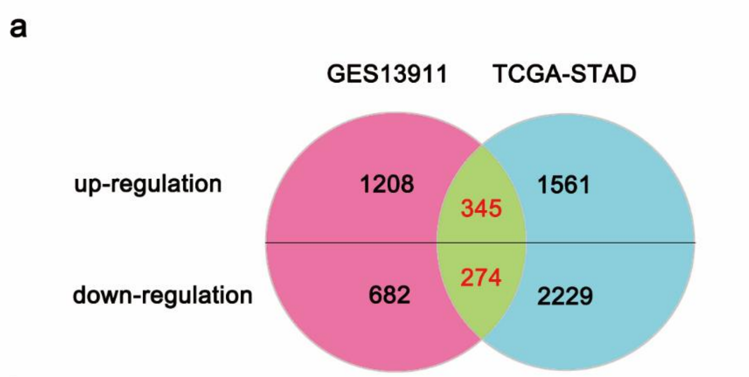

b

		GES13911		TCGA-STAD	
**GENECARD**	**RefSeq**	**Fold Change**	**P-value**	**Fold Change**	**P-value**
**CAPN1**	NM_001198868	UD		-1.19729	0.017923
**CAPN2**	NM_001146068	UD		-1.26171	0.006707
**CAPN3**	NM_000070	UD		1.240093	0.426301
**CAPN5**	NM_004055	-2.07876	0.000988	-1.15271	0.314865
**CAPN6**	NM_014289	-2.16138	0.001555	-2.62435	0.00262
**CAPN7**	NM_014296	UD		-1.08927	0.065896
**CAPN8**	NM_001143962	-6.97844	4.13E-08	3.038613	0.001241
**CAPN9**	NM_006615	-11.5379	3.37E-15	-3.14351	0.021873
**CAPN10**	NM_023083	UD		1.129645	0.172529
**CAPN11**	NM_007058	UD		1.677204	0.010713
**CAPN12**	NM_144691	UD		1.727806	0.001975
**CAPN13**	NM_144575	-3.50761	2.11E-14	-1.93066	0.259849
**CAPN14**	NM_001145122	UD		-2.44191	0.003297
**CAPNS1**	NM_001003962	UD		-1.33652	3.55E-05
**CAPNS2**	NM_032330	UD		-2.14082	0.004023

Supplementary Figure S1. Differentially expressed genes in GES13911 dataset and TCGA-STAD dataset. (**a**) Differentially expressed genes in GES13911 dataset (light red) and TCGA-STAD dataset (light blue) with area of overlap showing numbers of genes. (**b**) Differentially expressed members of calpain family in two datasets. UD, undetected.

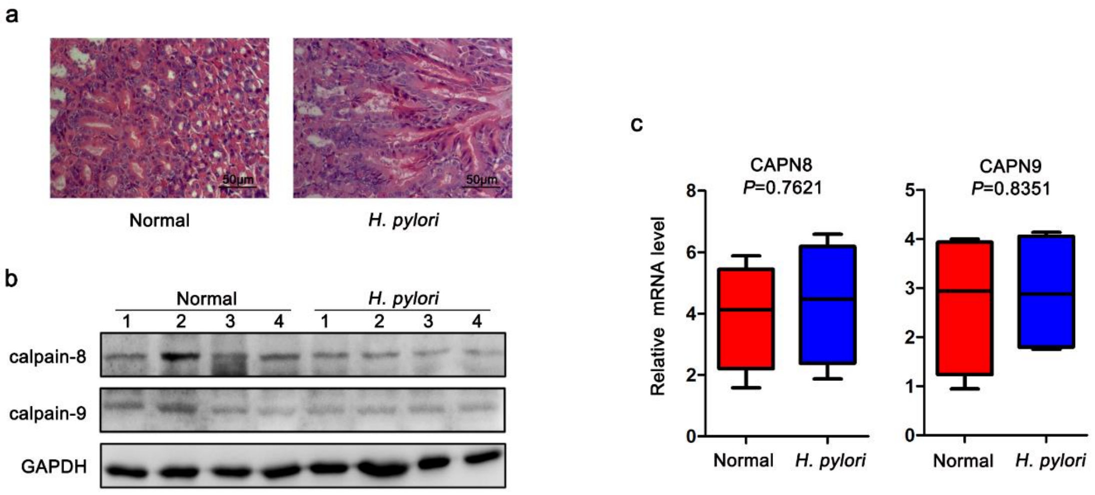

Supplementary Figure S2. The expression pattern of calpain-8 and -9 in mouse gastric mucosa with chronic *H. pylori* infection. C57BL/6 mice were inoculated intragastrically with saline control or *H. pylori* SS1 cell suspension. After 3 months, mice from both model group and control group were sacrificed and the stomachs were isolated from the mice for further experiment. (**a**) Representative HE staining in each group. (**b, c**) The protein and the mRNA levels of calpain-8 and calpain-9 in both groups were examined by western blot (**b**) and real-time PCR (**c**).

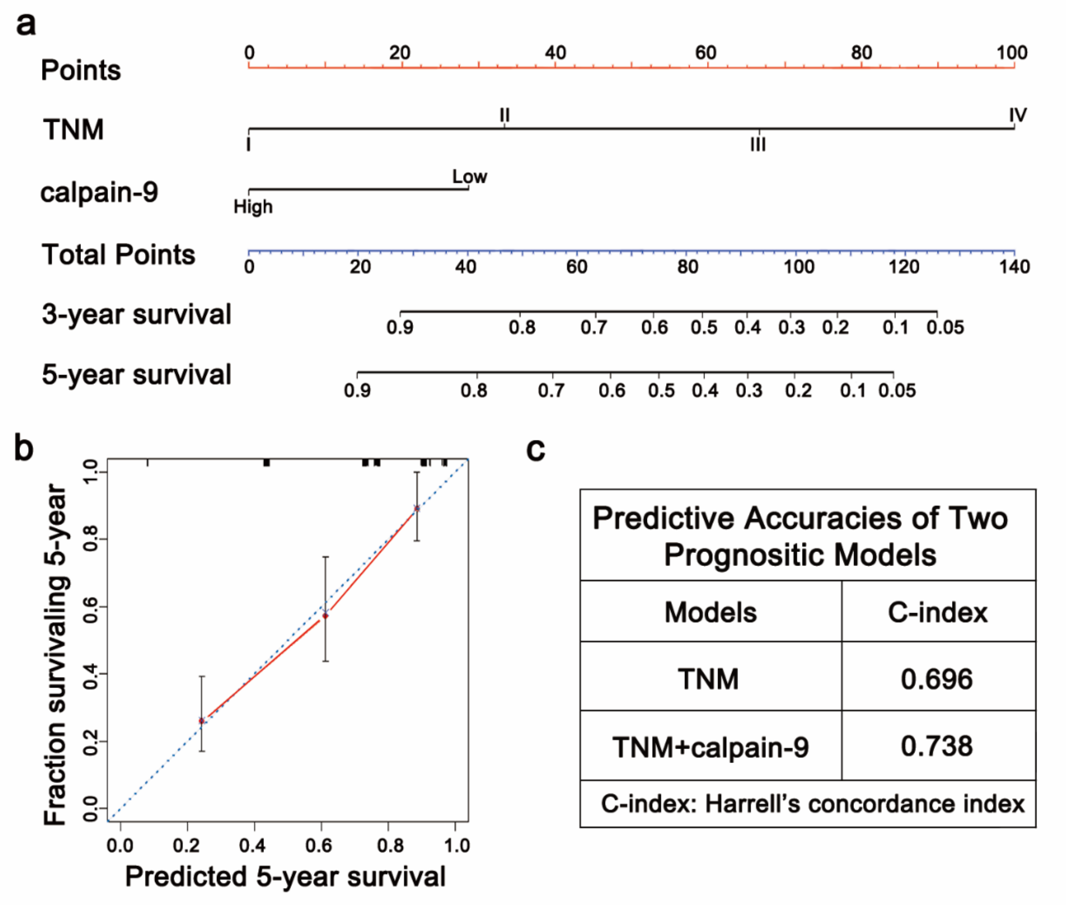

Supplementary Figure S3. Nomogram and calibration plot for the prediction of outcome in gastric cancer patients. (**a**) Nomogram for predicting clinical outcomes integrated calpain-9 expression (Low/High) with TNM classification (I/II/III/IV). (**b**) Calibration plot for nomogram predicted 5-year overall survival rate. The nomogram performed well with the ideal model. **(c**) The Harrell's concordance index (C-index) analysis for the generated nomogram or TNM stage alone.

Supplementary Table S1

CO-UPREGULATION		GES13911		TCGA-STAD				GES13911		TCGA-STAD	
GENECARD	RefSeq	Fold Change	P-value	Fold Change	P-value	GeneCard	RefSeq	Fold Change	P-value	Fold Change	P-value
**ABHD11**	NM_001145363	2.924214	9.42E-08	2.26832	1.43E-09	**CASC5**	NM_144508	3.230314	6.67E-06	5.401207	1.63E-14
**ADAMTS2**	NM_014244	6.736493	1.09E-09	4.219735	5.16E-07	**CBFB**	NM_001755	2.333418	1.4E-05	2.121174	7.39E-19
**ADAP1**	NM_001284308	2.505995	9.42E-07	2.258095	1.8E-10	**CCL15**	NM_004167	2.735488	0.017161	6.319715	1.54E-05
**AGMAT**	NM_024758	2.727213	0.000154	3.874587	3.3E-09	**CCL18**	NM_002988	2.825814	0.000146	2.822753	0.005543
**ANLN**	NM_001284301	4.292346	1.9E-07	4.916372	5.53E-21	**CCL20**	NM_001130046	2.198882	0.010381	4.402342	0.000641
**APOBEC3B**	NM_001270411	2.296684	0.000859	3.485333	7.95E-06	**CCL3**	NM_001001437	5.935226	7.59E-08	5.112738	5.37E-09
**APOC1**	NM_001645	4.134493	1.55E-06	13.47198	5.75E-12	**CCL4**	NM_002984	3.837024	2.92E-07	2.086886	0.001465
**APOE**	NM_000041	2.925817	0.000154	3.996079	1.02E-11	**CCNA2**	NM_001237	3.07355	5.32E-06	3.649698	4.4E-15
**AQP9**	NM_020980	2.016172	0.000191	2.728723	0.000995	**CCNB1**	NM_031966	4.275911	6.71E-07	3.503285	5.09E-16
**ARHGAP8**	NM_001017526	2.080339	2.16E-06	5.344696	2.99E-14	**CCNB2**	NM_004701	3.418729	1.96E-06	5.212058	5.42E-17
**ARSE**	NM_000047	2.967454	1.89E-05	3.704678	0.000673	**CCNE1**	NM_001238	2.884383	5.12E-05	3.524472	5.08E-06
**ASCL2**	NM_005170	3.034045	0.000334	5.350419	0.000177	**CCNE2**	NM_004702	3.115698	3.36E-07	2.765888	1.16E-09
**ASPM**	NM_001206846	8.232866	3.25E-08	8.70911	3.61E-20	**CDC20**	NM_001255	5.031787	1.31E-07	5.392867	1.03E-15
**ASPN**	NM_001193335	5.543612	5.23E-08	2.555708	0.002105	**CDC25B**	NM_001287516	4.237617	4.42E-09	2.541958	2.41E-13
**ATAD2**	NM_014109	4.176485	5.17E-10	3.858014	3.65E-20	**CDCA2**	NM_152562	3.245496	4.71E-07	8.003031	7.71E-17
**ATP11A**	NM_015205	3.53325	1.19E-09	3.342512	4.17E-23	**CDCA3**	NM_031299	2.884838	1.46E-06	2.469232	8.71E-09
**AURKA**	NM_003600	2.852832	8.92E-07	4.428368	3.62E-14	**CDCA5**	NM_080668	3.82866	4.34E-10	4.92196	1.28E-15
**AURKB**	NM_001256834	3.364727	5.34E-08	6.366631	4.61E-15	**CDCA7**	NM_031942	3.070157	8.33E-06	3.972676	9.42E-13
**BAMBI**	NM_012342	2.066666	0.010138	3.411141	0.000202	**CDCA8**	NM_001256875	2.620631	5.14E-07	4.687398	7.58E-17
**BATF2**	NM_138456	3.367119	3.47E-07	2.591482	4.37E-05	**CDH11**	NM_001797	2.472463	8.79E-06	2.483083	8.95E-07
**BCAT1**	NM_001178091	2.120737	0.000332	2.209944	0.000595	**CDH17**	NM_001144663	4.595644	0.020334	5.164161	0.002405
**BCL2A1**	NM_001114735	4.799473	5.49E-07	2.52237	3.47E-05	**CDK1**	NM_001130829	4.627426	6.49E-08	5.909384	2.57E-20

**BGN**	NM_001711	6.679762	8.94E-12	5.280199	1.16E-24	**CDKN3**	NM_001130851	4.643145	6.32E-07	3.289217	1.7E-11
**BID**	NM_001196	3.173162	4.76E-09	2.257933	1.9E-17	**CEACAM6**	NM_002483	4.389772	0.011388	3.253635	0.001418
**BIRC5**	NM_001012270	3.966814	9.81E-07	6.123536	4.76E-14	**CENPA**	NM_001042426	4.417323	7.48E-07	6.888127	2.57E-17
**BOP1**	NM_015201	3.414464	1.73E-08	2.103191	8.73E-06	**CENPF**	NM_016343	4.292358	3.34E-09	7.296919	2.71E-24
**BRIP1**	NM_032043	2.344197	2.73E-05	4.102425	2.56E-14	**CENPK**	NM_001267038	2.094702	0.000303	4.364635	1.03E-17
**BUB1**	NM_001278616	6.664866	1.33E-09	8.333946	9.72E-20	**CENPL**	NM_001127181	2.167	1.18E-08	2.196732	9.66E-15
**BUB1B**	NM_001211	5.443674	1.46E-08	7.549649	5.15E-19	**CENPN**	NM_001100624	3.27177	3.13E-07	2.016219	9.67E-11
**BYSL**	NM_004053	2.165501	1.56E-07	2.004422	3.49E-10	**CENPW**	NM_001012507	3.308516	2.4E-07	2.205129	3.14E-08
**C2**	NM_000063	2.385272	2.86E-05	3.086199	4.49E-10	**CEP55**	NM_001127182	4.790774	2.2E-08	7.198446	6.16E-21
**C2CD4A**	NM_207322	3.96148	9.8E-05	7.482272	1.02E-06	**CHD7**	NM_017780	2.070526	1.25E-08	2.013424	1.22E-11
**CAD**	NM_004341	2.728328	2.34E-09	2.208786	3.91E-18	**CHEK1**	NM_001114121	2.641073	2.16E-07	2.483052	3.97E-12
**CHI3L1**	NM_001276	13.06002	1.54E-10	6.449728	1.62E-06	**DKC1**	NM_001142463	2.381638	2.73E-08	2.319775	4.81E-21
**CKAP2**	NM_001098525	4.594348	3.08E-09	2.858082	1.05E-15	**DLEU2**	NR_002612	2.975944	1.07E-09	2.709766	1.27E-10
**CKS1B**	NM_001826	3.157957	1.8E-10	2.372106	2.17E-13	**DLGAP5**	NM_001146015	4.005429	5.3E-07	7.39488	5.16E-18
**CKS2**	NM_001827	4.040368	9.71E-09	3.62889	1.67E-15	**DNMT1**	NM_001130823	2.300448	6.03E-08	2.170021	8.38E-20
**CLDN1**	NM_021101	3.088305	5.95E-07	8.856234	2.34E-11	**DPY19L1**	NM_015283	2.060158	1.2E-05	2.41793	6.43E-15
**CLDN2**	NM_001171092	5.830947	8.41E-07	7.261114	0.000969	**DSCC1**	NM_024094	2.687043	5.24E-09	3.607865	3.33E-14
**CLDN3**	NM_001306	6.739877	0.000601	13.60762	1.19E-05	**DTL**	NM_001286229	3.646682	1.49E-07	5.35109	3.45E-18
**CLDN4**	NM_001305	3.445196	9.93E-06	5.672885	1.75E-17	**E2F3**	NM_001243076	3.026753	2.63E-10	2.523584	1.28E-20
**CLDN7**	NM_001185022	7.39496	1.56E-06	4.797796	9.35E-18	**E2F7**	NM_203394	3.022907	1.19E-05	8.079218	2.53E-16
**CLRN3**	NM_152311	3.734395	0.01334	7.80794	2.84E-05	**ECT2**	NM_001258315	4.035155	3.59E-09	5.670458	1.84E-27
**COL10A1**	NM_000493	5.20834	1.51E-06	91.867	1.69E-08	**EFNA2**	NM_001405	2.057359	0.024448	3.443249	0.001197
**COL11A1**	NM_001190709	5.968203	1.74E-06	71.32991	1.02E-07	**EFNA3**	NM_004952	2.650109	1.18E-05	2.852388	0.000137
**COL12A1**	NM_004370	5.7625	7.92E-11	3.188111	4.15E-12	**ENC1**	NM_001256574	3.261228	1.99E-06	4.484416	1.05E-27
**COL1A1**	NM_000088	2.240905	6.18E-09	7.582504	8.4E-31	**EPHB2**	NM_004442	2.957207	1.12E-06	7.488979	3.06E-11
**COL1A2**	NM_000089	3.215883	5.73E-07	3.595068	5.21E-16	**EPSTI1**	NM_001002264	3.754272	5.54E-08	2.327097	3.19E-06

**COL3A1**	NM_000090	2.599424	0.000109	3.940762	4.94E-17	**ETV4**	NM_001079675	2.0406	2.9E-05	8.286438	4.84E-10
**COL4A1**	NM_001845	3.528854	1.02E-12	3.109484	4.38E-22	**EZH2**	NM_001203247	3.450661	2.77E-07	3.932657	1.27E-16
**COL5A1**	NM_000093	2.797919	2.89E-06	2.882106	1.13E-11	**F2R**	NM_001992	2.545362	4.84E-06	2.91753	2.88E-17
**COL5A2**	NM_000393	4.217456	4.28E-10	3.880336	1.95E-18	**FAM60A**	NM_001135811	2.198259	4.17E-11	2.527959	3.68E-19
**COL6A3**	NM_004369	4.697924	1.13E-09	2.447632	1.52E-09	**FAM72A**	NM_001100910	6.468103	6.86E-08	9.663502	2.72E-23
**COL8A1**	NM_001850	17.61766	1.27E-11	3.297011	0.00152	**FAM83B**	NM_001010872	2.117077	0.000307	2.67038	8.8E-05
**CSE1L**	NM_001256135	3.429257	4.66E-10	2.095894	1.29E-16	**FAM83H**	NM_198488	2.399721	1.17E-07	3.892665	5.08E-18
**CST1**	NM_001898	6.323367	3.9E-06	231.2299	3.66E-08	**FANCD2**	NM_001018115	2.843492	4.69E-08	4.18265	1.34E-16
**CST4**	NM_001899	2.047271	2.53E-05	21.77252	1.2E-11	**FANCI**	NM_001113378	2.540645	1.2E-06	4.294069	2.25E-20
**CTHRC1**	NM_001256099	10.89702	6.35E-09	9.680546	6.85E-12	**FBXO5**	NM_001142522	3.108851	2.7E-07	2.012996	1.85E-10
**CXCL1**	NM_001511	5.082205	3.63E-07	8.322332	3.64E-07	**FCGR2A**	NM_001136219	2.727427	1.72E-05	2.213804	1.02E-06
**CXCL10**	NM_001565	8.330608	2.79E-08	4.4084	5.07E-05	**FCGR3A**	NM_000569	4.617561	6.23E-06	4.9443	7.4E-09
**CXCL11**	NM_005409	4.757898	4.16E-05	5.902119	2.81E-06	**FEN1**	NM_004111	2.778399	8.21E-08	2.107377	8.47E-13
**CXCL16**	NM_001100812	2.3281	2.3E-05	2.351059	3.03E-14	**FERMT1**	NM_017671	2.040345	3.92E-05	3.532326	3.62E-12
**CXCL9**	NM_002416	9.40643	2.03E-07	5.664222	2.39E-06	**FIGNL1**	NM_001042762	3.852637	2.94E-08	2.187696	2.88E-10
**DBF4**	NM_006716	2.969358	4.35E-09	3.253791	1.23E-16	**FMNL2**	NM_001004417	2.429822	1.33E-10	2.208198	2.51E-11
**DCAF13**	NM_015420	2.477539	6.48E-12	2.213989	7.34E-15	**FN1**	NM_002026	6.707603	1.71E-10	2.380318	3.83E-06
**DDX21**	NM_001256910	2.351493	5.59E-10	2.148692	1.26E-19	**FNDC1**	NM_032532	9.524289	7.42E-09	11.24006	3.57E-06
**DEPDC1**	NM_001114120	4.098693	2.49E-07	5.594769	1.53E-14	**FOXC1**	NM_001453	2.262347	2.68E-05	2.168476	0.002759
**DEPDC1B**	NM_001145208	6.434735	2.58E-08	5.793657	7.77E-16	**GAS2L3**	NM_174942	2.614119	0.000116	2.041285	9.82E-06
**GBP5**	NM_001134486	3.134768	0.000171	2.894456	0.000209	**KIF18B**	NM_001080443	4.148463	3.56E-09	11.01718	1.85E-19
**GDF15**	NM_004864	6.18039	1.22E-08	11.0361	5.68E-13	**KIF20A**	NM_005733	3.747087	2.84E-08	6.861093	6.46E-19
**GDPD5**	NM_030792	2.145985	2.18E-05	3.255481	9.9E-08	**KIF20B**	NM_001284259	2.144407	1.22E-08	2.724888	1.67E-16
**GGH**	NM_003878	4.604421	2.96E-09	2.875953	2.03E-06	**KIF23**	NM_001281301	5.251066	3.72E-09	6.352478	1.13E-19
**GINS1**	NM_021067	4.445216	1.63E-07	4.283423	6.49E-15	**KIF2C**	NM_006845	5.75107	9.92E-10	4.969033	1.49E-15
**GINS2**	NM_016095	3.536195	5.19E-07	2.618943	2.65E-08	**KIF4A**	NM_012310	4.089203	5.5E-08	7.305185	2.96E-17

**GPRC5A**	NM_003979	3.730644	1.66E-05	4.095404	2.16E-12	**KPNA2**	NM_002266	2.366603	1.13E-07	2.694287	2.81E-21
**GTPBP4**	NM_012341	2.386963	8.95E-08	2.02624	7.91E-17	**KRT7**	NM_005556	3.066259	0.001323	5.346349	0.000973
**GTSE1**	NM_016426	2.242315	7.86E-08	5.982458	6.03E-17	**LAMP3**	NM_014398	2.925608	5.14E-06	4.323844	6.24E-10
**GZMB**	NM_004131	2.121942	0.005893	2.003763	0.004749	**LEF1**	NM_001130713	2.511854	1.02E-05	2.654133	1.03E-08
**HEATR1**	NM_018072	2.711348	2.07E-10	2.207646	8.46E-21	**LGR5**	NM_001277226	3.882192	8.86E-06	6.600057	0.000257
**HELLS**	NM_001289067	2.133923	1.55E-05	5.624619	1.62E-20	**LIF**	NM_001257135	2.600416	1.1E-06	4.76182	6.54E-11
**HJURP**	NM_001282962	3.024597	5.63E-07	8.253621	4.16E-18	**LIPG**	NM_006033	3.020232	4.95E-06	4.878578	1.07E-08
**HMGA1**	NM_002131	2.323181	1.88E-08	2.323776	3.17E-10	**LMNB1**	NM_001198557	2.684858	2.33E-05	3.27753	4.86E-18
**HMGB3**	NM_005342	3.232659	1.35E-10	3.366064	4.16E-15	**LOX**	NM_001178102	2.621134	6.48E-05	2.255853	1.42E-05
**HMMR**	NM_001142556	3.861096	5.33E-07	7.900792	3.1E-18	**LOXL2**	NM_002318	2.979989	1.06E-07	2.641822	8.08E-12
**HOXA10**	NM_018951	7.526424	4.98E-07	21.79083	2.58E-07	**LY6E**	NM_001127213	7.040238	1.45E-10	2.326483	7.96E-09
**HOXB6**	NM_018952	2.045351	0.000189	2.892711	0.000231	**MACC1**	NM_182762	2.032916	0.007213	4.215677	1.14E-11
**HOXB7**	NM_004502	6.697906	9.29E-09	2.714296	5.79E-05	**MAD2L1**	NM_002358	2.507857	4.98E-06	3.704309	3.55E-13
**HOXC6**	NM_004503	6.435511	5.95E-09	5.8771	1.38E-07	**MAGEA3**	NM_005362	6.1883	0.000108	15.1235	0.000699
**HPSE**	NM_001098540	3.825021	4.46E-07	2.502198	3.32E-06	**MAGEA6**	NM_005363	6.816183	0.000105	16.0619	0.000778
**HSPD1**	NM_002156	3.051192	2.88E-05	2.430965	1.01E-20	**MARVELD3**	NM_001017967	2.769418	1.87E-05	3.997069	7.86E-11
**HSPH1**	NM_001286503	2.171233	0.000139	2.160286	1.21E-12	**MASTL**	NM_001172303	2.552586	3.98E-08	2.028942	6.03E-12
**IFI30**	NM_005027	2.506851	3.61E-08	2.962242	3.92E-17	**MCM2**	NM_004526	3.965328	7.17E-10	2.781493	1.19E-15
**IFI44L**	NM_006820	3.085477	0.00016	2.087325	0.020361	**MCM4**	NM_005914	2.636135	1.25E-06	2.643379	4.18E-15
**IFI6**	NM_002038	4.704781	2.14E-07	2.728621	2.61E-07	**MCM7**	NM_001278595	2.570267	1.34E-08	2.096013	4.65E-12
**IGF2BP2**	NM_001007225	2.288758	0.000108	4.278749	7.38E-12	**MCM8**	NM_001281520	2.779435	2.74E-07	2.429955	3.26E-11
**IL32**	NM_001012631	4.479348	7.78E-09	3.341924	5.45E-13	**MDK**	NM_001012333	3.030734	7.95E-05	2.539128	3.33E-10
**INHBA**	NM_002192	5.970443	1.63E-08	12.66557	1.13E-12	**MELK**	NM_001256685	3.52127	2.49E-06	6.98419	3.19E-17
**IQGAP3**	NM_178229	3.054664	6.9E-06	7.849868	9.06E-23	**MEST**	NM_001253900	5.680621	4.83E-08	4.336111	1.35E-13
**ITGA2**	NM_002203	3.628106	4.83E-06	2.384338	2.54E-08	**MET**	NM_000245	2.258564	5.23E-07	3.493078	3.57E-15
**KIAA0101**	NM_001029989	2.525061	3.29E-05	4.999128	1.02E-15	**MFAP2**	NM_001135247	9.927048	2.84E-11	4.024076	2.21E-06

**KIF11**	NM_004523	3.420278	4.26E-06	4.425147	3.39E-22	**MICB**	NM_001289160	2.917423	3.32E-05	2.887183	3.12E-08
**KIF14**	NM_014875	5.166789	1.1E-09	11.2867	3.03E-21	**MKI67**	NM_001145966	2.540273	7.07E-05	7.551701	3.96E-36
**KIF15**	NM_020242	2.482955	2.44E-08	5.715718	9.16E-16	**MMP1**	NM_001145938	2.407264	1.09E-05	10.15696	1.52E-06
**MMP12**	NM_002426	9.94366	1E-07	10.68756	3.53E-05	**PARP14**	NM_017554	2.432076	3.95E-08	2.302106	3.54E-16
**MMP3**	NM_002422	7.101636	1.85E-07	18.1766	5.36E-05	**PBK**	NM_001278945	4.029626	5.35E-07	6.713036	1.09E-13
**MMP7**	NM_002423	4.363455	6.01E-06	15.01665	0.000173	**PCNA**	NM_002592	2.418145	1.51E-07	2.260265	3.83E-15
**MMP9**	NM_004994	3.515196	1.42E-06	5.879531	2.53E-08	**PDGFRB**	NM_002609	2.047991	2.7E-06	2.526703	1.36E-11
**MSH2**	NM_000251	3.59782	4.21E-11	2.147993	1.7E-12	**PGM2L1**	NM_173582	2.105012	2.51E-05	3.182025	2.94E-15
**MSLN**	NM_001177355	3.632165	0.000105	11.96777	0.001159	**PIK3AP1**	NM_152309	3.592364	1.09E-07	3.884954	1.3E-15
**MSR1**	NM_002445	2.290772	1.29E-05	3.659696	9.05E-07	**PKDCC**	NM_138370	5.792373	2.08E-06	2.569408	4.83E-05
**MSX2**	NM_002449	2.017411	0.005027	3.237062	6.58E-06	**PLA2G7**	NM_001168357	5.818132	4.62E-09	9.132269	1.45E-14
**MXRA5**	NM_015419	2.043933	0.00054	2.695675	2.3E-10	**PLAU**	NM_001145031	6.564345	6.07E-11	3.178416	4.66E-11
**MYB**	NM_001130172	3.390553	9.09E-06	5.622652	1.53E-08	**PLAUR**	NM_001005376	2.428433	0.000125	2.396088	4.64E-09
**MYBL2**	NM_001278610	2.718004	7.68E-07	9.68512	2.81E-21	**PLOD3**	NM_001084	2.894064	9.5E-11	2.537126	8.54E-18
**MYO1B**	NM_001130158	2.191995	1.61E-06	2.301705	2.48E-14	**PLXNA1**	NM_032242	2.390765	4.85E-09	2.096486	1.07E-14
**NCAPG**	NM_022346	2.582104	4.15E-07	7.267725	1.37E-18	**PMAIP1**	NM_021127	3.332206	1.82E-06	2.535922	1.7E-06
**NCAPG2**	NM_001281932	2.247396	4.88E-06	2.78924	1.9E-13	**PMEPA1**	NM_001255976	3.958864	6.01E-08	3.367255	9.13E-12
**NCAPH**	NM_001281710	2.003055	3.87E-07	5.685757	7.33E-16	**PNPT1**	NM_033109	2.400742	1.32E-10	2.041405	3.38E-14
**NDC80**	NM_006101	4.469064	6.89E-08	5.119332	6.46E-18	**PODXL**	NM_001018111	2.725621	1.93E-10	2.473951	1.36E-17
**NEK2**	NM_001204182	7.669709	9.57E-09	7.974781	2E-17	**PPAT**	NM_002703	2.311421	5.63E-06	2.508939	1.18E-12
**NFE2L3**	NM_004289	6.951775	9.04E-12	5.796152	1.64E-25	**PPP1R1B**	NM_001242464	3.930879	0.000732	2.776722	0.011245
**NID2**	NM_007361	4.886446	1.05E-10	2.531933	5.14E-07	**PRC1**	NM_001267580	6.061792	4.74E-08	3.689038	1.32E-18
**NME1**	NM_000269	3.120823	1.7E-09	2.200601	2.26E-12	**PRIM1**	NM_000946	2.174002	5.21E-07	2.051322	3.35E-09
**NOP2**	NM_001033714	2.701912	1.05E-09	2.229895	1.12E-16	**PRIM2**	NM_000947	2.168527	1.49E-08	2.095114	6.07E-15
**NOTCH3**	NM_000435	2.719378	3.57E-08	2.237939	3.88E-10	**PRKDC**	NM_001081640	2.310228	1.46E-08	2.118558	4.33E-18
**NUDCD1**	NM_001128211	4.352376	4.54E-13	2.385651	3.39E-15	**PRR11**	NM_018304	4.312383	4.53E-07	3.483218	1.32E-14

**NUF2**	NM_031423	6.156864	1.83E-09	7.317485	1.61E-18	**PSRC1**	NM_001005290	3.216516	1.12E-07	2.158656	5.92E-07
**NUP107**	NM_020401	2.410373	5.28E-08	2.122707	1.54E-12	**PTTG1**	NM_001282382	2.978406	6.4E-07	2.832096	7.22E-11
**NUSAP1**	NM_001129897	3.469398	1.67E-06	4.461899	3.46E-21	**PUS7**	NM_019042	3.952763	3.77E-10	2.813347	1.95E-13
**OAS3**	NM_006187	2.067578	5.99E-05	2.217927	1.47E-09	**RAD51**	NM_001164269	2.253736	1.78E-06	3.735777	1.11E-16
**OIP5**	NM_007280	4.12922	1.4E-07	3.755546	3.96E-14	**RAD51AP1**	NM_001130862	3.826546	3.67E-07	4.532566	2.03E-16
**OLFML2B**	NM_015441	3.508141	5.06E-07	3.548199	3.57E-07	**RARRES1**	NM_002888	6.773479	1.62E-05	2.460981	0.002579
**ONECUT2**	NM_004852	2.028773	0.035025	10.89616	6.72E-08	**RBL1**	NM_002895	2.421893	9.49E-08	2.253062	1.73E-10
**OSBPL3**	NM_015550	2.300056	1.75E-06	3.041186	1.56E-22	**RCC1**	NM_001048194	2.851565	1.1E-07	7.054448	3.27E-14
**OSMR**	NM_001168355	3.037355	1.16E-06	2.007331	0.000158	**RCC2**	NM_001136204	2.394175	6.73E-11	2.569875	6.51E-30
**OVOL1**	NM_004561	2.292996	0.000422	3.443731	0.00027	**RCN3**	NM_020650	2.640665	1.05E-05	2.170628	4.13E-05
**P4HA1**	NM_000917	2.191808	1.96E-05	2.005728	1.18E-08	**RFC3**	NM_002915	3.042734	5.33E-07	3.13757	1.9E-14
**PAFAH1B3**	NM_001145939	2.154829	9.88E-08	2.115959	9.33E-10	**RFC4**	NM_002916	2.957937	2.17E-07	2.115899	8.6E-12
**RIPK2**	NM_003821	2.505711	3.21E-08	2.370388	1E-14	**TFAP2A**	NM_001032280	4.457445	1.77E-07	3.118733	0.000553
**RPP25**	NM_017793	2.226238	3.39E-06	2.252384	1.23E-11	**TGIF1**	NM_001278682	3.225332	1.85E-14	2.016603	3.49E-17
**RRM2**	NM_001034	3.167225	1.09E-05	5.214598	3.97E-21	**THBS2**	NM_003247	11.05046	6.37E-10	5.343186	1.97E-08
**RTKN**	NM_001015055	4.827374	4.99E-13	2.14348	2.34E-11	**THY1**	NM_006288	6.141903	6.4E-10	3.338926	9.11E-14
**SCML1**	NM_001037535	2.445854	3.51E-07	2.162547	3.32E-05	**TIGD1**	NM_145702	2.105354	2.75E-07	2.241432	3.97E-10
**SERPINA1**	NM_000295	2.012828	0.007314	3.547525	2.26E-06	**TIMELESS**	NM_003920	2.062772	1.62E-07	3.126608	2.44E-18
**SERPINB5**	NM_002639	5.09555	0.002027	3.975475	0.000539	**TIMP1**	NM_003254	4.305043	5.09E-09	2.708027	8.43E-15
**SERPINE1**	NM_000602	2.138746	0.00048	3.596301	5.35E-08	**TMEM206**	NM_001198862	3.096153	1.15E-11	2.202759	6.05E-17
**SERPINH1**	NM_001207014	10.94461	1.99E-14	2.605328	3.75E-22	**TNFRSF10B**	NM_003842	2.084648	1.94E-07	2.50778	3.66E-17
**SGOL2**	NM_001160033	2.891123	1.25E-06	3.351256	2.54E-15	**TNFRSF12A**	NM_016639	4.61076	6.2E-11	2.602693	1.24E-08
**SHCBP1**	NM_024745	4.134201	1.91E-06	4.136634	3.11E-17	**TNS4**	NM_032865	2.411695	1.99E-05	4.143315	0.003855
**SKP2**	NM_001243120	3.162955	9.52E-08	2.013993	5.54E-07	**TOP1MT**	NM_001258446	2.135833	2.2E-07	2.008539	1.29E-08
**SLAMF8**	NM_020125	2.692191	5.69E-05	2.530798	3.6E-06	**TOP2A**	NM_001067	7.316677	9.58E-09	7.553393	2.76E-26
**SLC12A8**	NM_001195483	2.020391	4.61E-05	3.770903	5.31E-18	**TPX2**	NM_012112	5.998195	3.08E-09	7.804894	3.07E-27

**SLC27A2**	NM_001159629	2.219131	0.002824	2.987176	5.56E-06	**TRIB3**	NM_021158	4.462335	2.64E-07	3.239358	4.36E-05
**SLC39A10**	NM_001127257	6.071806	9.71E-13	3.270419	3.56E-15	**TRIM29**	NM_012101	4.831126	0.000114	3.044135	0.024307
**SLC4A11**	NM_001174089	2.163623	0.00033	6.708109	1.95E-07	**TRIM59**	NM_173084	3.480883	1.62E-07	2.793015	5.64E-15
**SLC5A6**	NM_021095	2.914039	2.68E-08	2.472069	4.47E-15	**TRIP13**	NM_001166260	5.729864	3.06E-10	5.302314	4.02E-15
**SMC2**	NM_001042550	2.706622	1.21E-07	2.179047	1.51E-13	**TTF2**	NM_003594	2.211342	2.7E-07	2.102979	1.2E-12
**SMC4**	NM_001002799	4.774077	1.22E-08	2.198603	1.21E-15	**TTK**	NM_001166691	3.476793	1.5E-06	8.07131	6.23E-16
**SNRPD1**	NM_001291916	2.475085	2.92E-09	2.091377	1.44E-11	**TTYH3**	NM_025250	2.171812	6.08E-07	3.476042	6.89E-25
**SNX10**	NM_001199835	6.708859	6.22E-14	3.768813	2.15E-13	**TYMS**	NM_001071	3.904704	1.5E-06	2.925891	2.37E-13
**SOX4**	NM_003107	2.157755	1.92E-06	3.617949	5.06E-24	**UBE2C**	NM_001281741	5.830752	4.83E-09	7.777198	2.22E-17
**SPAG5**	NM_006461	2.07964	3.45E-05	4.599277	3.66E-17	**UBE2S**	NM_014501	4.386306	2.34E-07	2.311105	1.24E-07
**SPARC**	NM_003118	4.443717	1.09E-11	2.59221	2.15E-13	**UBE2T**	NM_014176	4.397908	9.33E-10	4.19151	2.1E-14
**SPC25**	NM_020675	4.920783	6.97E-08	4.902079	7.37E-17	**UHRF1**	NM_001048201	2.985883	1.62E-07	5.735354	1.6E-15
**SPP1**	NM_000582	22.33105	7.47E-12	10.04125	3.7E-07	**VCAN**	NM_001126336	2.27073	6.13E-05	3.282164	2.27E-12
**STAMBPL1**	NM_020799	2.711159	6.86E-08	2.18027	1.81E-08	**VIL1**	NM_007127	2.408168	0.040035	12.06078	1.63E-07
**STAT1**	NM_007315	2.116493	1.37E-06	2.170138	6.1E-13	**WDHD1**	NM_001008396	2.192082	1.5E-06	3.043404	7.34E-14
**STIL**	NM_001048166	3.894994	7.3E-11	5.877928	9.4E-19	**WDR72**	NM_001277176	12.13917	1.76E-06	11.77791	0.000571
**STMN1**	NM_001145454	2.632088	8.23E-08	2.059349	2.31E-12	**XPO5**	NM_020750	3.021366	8.21E-09	2.220044	7.1E-15
**SULF1**	NM_001128204	6.064707	2.01E-08	5.001276	2.28E-12	**ZIC2**	NM_007129	6.906656	4.98E-08	10.28338	8.3E-05
**SUV39H2**	NM_001193424	3.365931	1.78E-08	2.000737	5.71E-11	**ZNF367**	NM_153695	2.816927	2.81E-06	2.841867	1.12E-13
**TACC3**	NM_006342	2.972641	9.87E-09	2.887771	9.48E-16	**ZWILCH**	NM_001287821	3.293512	2.4E-07	2.810391	1.97E-19
**TEAD4**	NM_003213	2.703665	3.16E-10	2.761347	4.2E-14						

CO-DOWNREGULATION		GES13911		TCGA-STAD				GES13911		TCGA-STAD	
**GENECARD**	**RefSeq**	**Fold Change**	**P-value**	**Fold Change**	**P-value**	**GeneCard**	**RefSeq**	**Fold Change**	**P-value**	**Fold Change**	**P-value**
**AADAC**	NM_001086	-8.31858	5.95E-09	-3.34642	0.004212	**BEX4**	NM_001080425	-2.13716	0.000439	-3.56223	5.29E-22
**ABAT**	NM_000663	-2.1599	0.006995	-2.34964	3.51E-08	**BEX5**	NM_001012978	-2.00597	0.000866	-3.10366	7.35E-06
**ABCA8**	NM_001288985	-13.1132	8.51E-11	-11.2914	3.13E-26	**BNIP3**	NM_004052	-3.42983	1.93E-05	-2.71504	4.14E-12
**ABI3BP**	NM_015429	-2.11941	0.006633	-5.74518	3.26E-14	**C16orf89**	NM_001098514	-15.9323	3.16E-12	-22.6547	1.3E-44
**ACACB**	NM_001093	-2.70331	3.32E-10	-3.18054	8.82E-17	**C2orf40**	NM_032411	-4.2101	3.3E-06	-38.8418	2.43E-47
**ACE2**	NM_021804	-2.46972	0.021968	-3.54253	0.000238	**C6orf58**	NM_001010905	-20.2959	1.27E-07	-9.27765	5.2E-06
**ADAMTS1**	NM_006988	-2.08559	0.000838	-3.33594	4.87E-15	**C7**	NM_000587	-2.42029	0.007957	-11.3529	1.32E-15
**ADAMTS15**	NM_139055	-2.48208	6.55E-08	-3.14548	3.44E-08	**CA2**	NM_000067	-7.67873	5.55E-07	-2.47499	0.001866
**ADH1B**	NM_000668	-7.86817	1.45E-07	-16.9078	4.19E-30	**CACNA2D1**	NM_000722	-2.21288	9.94E-05	-2.28437	7.48E-05
**ADH1C**	NM_000669	-12.4798	1.11E-08	-3.57858	0.002187	**CAPN6**	NM_014289	-2.16138	0.001555	-2.62435	0.00262
**ADH4**	NM_000670	-2.63191	0.027275	-5.33086	6.95E-06	**CAPN9**	**NM_006615**	**-11.5379**	**3.37E-15**	**-3.14351**	**0.021873**
**AFF3**	NM_001025108	-2.90149	1.05E-05	-6.8311	4.3E-29	**CBR1**	NM_001286789	-2.20401	0.000239	-3.94001	1.86E-28
**AGPAT9**	NM_001256421	-4.46943	2.62E-08	-3.58614	6.48E-18	**CCKBR**	NM_176875	-11.5455	8.21E-10	-20.4782	1.1E-22
**AKR1B10**	NM_020299	-18.083	2.61E-08	-8.302	1.07E-07	**CCL11**	NM_002986	-2.75761	0.000752	-2.05106	0.005667
**AKR1C1**	NM_001135241	-7.70076	6.6E-12	-8.83551	2.17E-24	**CCL14**	NM_004166	-2.39644	0.000206	-11.3338	7.71E-43
**AKR1C2**	NM_001135241	-10.3085	1.14E-07	-10.7309	2.08E-31	**CD36**	NM_000072	-3.49605	1.11E-07	-4.24641	3.91E-20
**AKR1C3**	NM_001253908	-4.9491	0.00011	-2.09832	0.002166	**CFD**	NM_001928	-2.67943	1.11E-06	-9.24585	3.58E-37
**ALDH1A1**	NM_000689	-3.72657	5.62E-06	-2.02869	0.006561	**CHGA**	NM_001275	-26.167	2.03E-13	-26.1404	3.95E-19
**ALDH3A1**	NM_000691	-10.6771	1.8E-08	-5.93609	2.11E-09	**CHIA**	NM_001040623	-14.642	1.57E-06	-8.91147	4.16E-15
**ALDH6A1**	NM_001278593	-2.60907	8.68E-08	-2.31264	1.24E-21	**CHRM3**	NM_000740	-4.31226	3.38E-06	-2.36645	2.63E-05
**ALDOB**	NM_000035	-4.16009	0.001405	-3.81058	0.001123	**CIDEB**	NM_014430	-2.10953	0.00179	-3.26995	6.95E-29
**ANKRD29**	NM_173505	-2.65133	0.000166	-2.88818	1.57E-09	**CIDEC**	NM_001199551	-2.5532	0.000167	-5.62872	2.24E-07
**APLP1**	NM_001024807	-3.0495	1.23E-07	-2.53312	0.000105	**CKB**	NM_001823	-5.26041	1.37E-08	-6.83082	1.03E-16
**APOA4**	NM_000482	-2.0576	0.004696	-23.7343	2.17E-12	**CKMT2**	NM_001099735	-4.21653	2.11E-07	-24.0069	2.49E-46

**APOB**	NM_000384	-3.29696	0.020458	-15.2855	3.13E-15	**CLCA1**	NM_001285	-7.10255	0.000315	-13.6668	3.06E-09
**APOBEC2**	NM_006789	-3.20627	3.8E-08	-6.34746	1.08E-21	**CLEC3B**	NM_003278	-3.40337	1.09E-06	-11.2359	2.45E-69
**APOD**	NM_001647	-2.71822	0.001149	-3.07433	9.01E-05	**CLU**	NM_001831	-4.03853	7.46E-05	-4.012	1.14E-08
**AQP4**	NM_001650	-35.7272	3.79E-10	-37.6695	1.57E-44	**COL4A5**	NM_000495	-3.68259	8.78E-09	-5.06831	4.95E-16
**ARHGEF37**	NM_001001669	-3.81076	1.29E-09	-3.41398	6.83E-24	**CPA2**	NM_001869	-23.404	1.3E-09	-15.293	7.25E-10
**ATP4A**	NM_000704	-123.777	7.57E-13	-59.0173	8.26E-20	**CPA3**	NM_001870	-4.29192	5.42E-07	-3.37921	2.52E-08
**ATP4B**	NM_000705	-348.129	1.74E-14	-55.7655	4.6E-22	**CPE**	NM_001873	-3.26049	9.85E-05	-4.12633	1.11E-16
**B3GNT6**	NM_138706	-2.25353	8.45E-10	-11.3305	1.97E-11	**CSTA**	NM_005213	-4.08637	2.91E-06	-5.35791	1.92E-13
**BEX2**	NM_001168399	-2.91545	0.000263	-6.30129	1E-11	**CWH43**	NM_001286791	-7.06837	4.99E-11	-63.8313	8.54E-45
**CXCL12**	NM_000609	-2.75112	0.002649	-3.23928	3.34E-12	**FAM189A2**	NM_001127608	-3.47232	4.81E-08	-5.28456	1.88E-14
**CXCL14**	NM_004887	-4.34174	2.61E-05	-3.18743	3.86E-05	**FAM3B**	NM_058186	-15.626	1.74E-08	-3.90674	0.000224
**CXCL17**	NM_198477	-13.0187	6.61E-09	-7.5035	1.05E-07	**FCGBP**	NM_003890	-14.7897	6.48E-09	-2.89605	0.00283
**CYBRD1**	NM_001127383	-2.12458	0.000488	-3.42737	2.07E-12	**FHL1**	NM_001159699	-2.99677	1.36E-05	-8.45654	7.91E-19
**CYP2C18**	NM_000772	-7.74588	3.83E-07	-2.90239	0.003397	**FRMD3**	NM_001244959	-2.04089	0.000228	-2.48449	2.9E-07
**CYP3A4**	NM_001202855	-2.96949	0.000127	-8.03134	3.03E-08	**GABARAPL1**	NM_031412	-2.16936	7.31E-06	-2.24952	3.09E-13
**CYP4F12**	NM_023944	-2.78495	1.68E-06	-3.00789	5.38E-05	**GATA5**	NM_080473	-2.15803	1.16E-06	-4.30451	0.001311
**DEFA5**	NM_021010	-10.3345	0.000515	-23.4977	1.75E-13	**GBA3**	NM_001128432	-3.5258	0.015182	-4.63224	5.32E-05
**DEFA6**	NM_001926	-8.03334	0.000369	-11.8881	7.07E-09	**GCNT2**	NM_001491	-5.04706	5.67E-09	-2.51784	1.04E-09
**DEFB4A**	NM_001205266	-4.95531	8.3E-07	-6.3077	7.99E-09	**GDPD3**	NM_024307	-3.63968	1.11E-05	-3.03927	8.69E-08
**DHRS7**	NM_016029	-2.46798	1.59E-08	-2.10627	1.99E-15	**GGT6**	NM_001122890	-2.136	0.010347	-3.47778	0.001244
**DHRS9**	NM_001142270	-2.38535	0.003003	-3.07841	1.6E-05	**GHRL**	NM_001134941	-10.7241	3.48E-10	-7.76167	1.63E-11
**DISP1**	NM_032890	-2.27532	8.48E-08	-2.10972	3.27E-17	**GIF**	NM_005142	-340.593	9.29E-14	-17.9699	4.17E-08
**DMD**	NM_000109	-3.81954	1.39E-07	-3.73537	2.32E-13	**GKN1**	NM_019617	-60.8172	2.25E-08	-107.57	3.71E-23
**DNER**	NM_139072	-5.01365	3.49E-05	-12.4718	1.4E-21	**GKN2**	NM_182536	-78.8457	1.16E-09	-63.3942	3.28E-19
**DPCR1**	NM_080870	-18.3119	2.93E-07	-6.10049	3.24E-05	**GLUL**	NM_001033044	-2.45837	0.000318	-2.04787	1.18E-13
**DPT**	NM_001937	-4.28776	1.17E-07	-23.6541	5.85E-61	**GNG7**	NM_052847	-2.53433	1.06E-07	-7.16059	1.24E-50

**DUOX1**	NM_017434	-2.69015	6.29E-05	-2.43867	0.000502	**GPD1L**	NM_015141	-2.64304	5.49E-11	-2.03648	2.27E-14
**DUOXA2**	NM_207581	-3.9807	1.88E-07	-2.73483	0.04896	**GPR155**	NM_001033045	-17.5528	1.31E-09	-3.5787	4.42E-18
**DUSP5**	NM_004419	-2.0999	0.003647	-3.2379	1.85E-16	**GPR64**	NM_001079858	-5.0085	1.21E-08	-3.31015	3.05E-08
**ECHDC2**	NM_001198961	-2.20551	1.11E-06	-2.42885	3.47E-15	**GPX3**	NM_002084	-5.48352	1.42E-08	-7.75411	3.16E-33
**EDIL3**	NM_001278642	-2.06806	8.95E-05	-2.14931	1.53E-07	**GSTA1**	NM_145740	-6.00365	3.1E-07	-4.18689	0.002556
**EGLN3**	NM_022073	-2.00798	0.001223	-2.00931	4.15E-08	**GSTA4**	NM_001512	-3.14828	7.13E-06	-2.34866	2.72E-11
**ELL2**	NM_012081	-2.82273	6.68E-10	-2.63163	1.01E-21	**HADH**	NM_001184705	-2.23127	6.16E-10	-2.11484	3.41E-18
**ENTPD3**	NM_001248	-2.19021	0.000522	-7.68791	7.3E-22	**HHIP**	NM_022475	-6.51118	3.61E-11	-2.27129	0.024363
**ENTPD5**	NM_001249	-2.80053	5.33E-07	-2.13352	4.2E-08	**HOMER2**	NM_004839	-3.5011	2.96E-07	-6.39411	1.35E-15
**ESRRG**	NM_001134285	-50.7001	1.62E-12	-20.6028	1.82E-40	**HPGD**	NM_000860	-13.1148	3.03E-09	-8.42964	7.85E-20
**ETFDH**	NM_001281737	-2.65619	2.47E-11	-2.43848	1.76E-24	**HSD17B6**	NM_003725	-2.12479	0.000319	-2.7629	7.69E-12
**EYA2**	NM_005244	-2.98154	1.25E-05	-3.5624	3.04E-05	**HSPB8**	NM_014365	-2.00496	0.000712	-8.79613	1.04E-26
**F13A1**	NM_000129	-3.90991	1.54E-06	-4.44138	1.15E-12	**ID3**	NM_002167	-2.08693	5.97E-07	-2.06508	3.55E-11
**FABP4**	NM_001442	-4.23952	0.000312	-10.9664	1.64E-28	**ID4**	NM_001546	-6.91243	1.77E-10	-3.92844	5.84E-25
**FAM107A**	NM_001076778	-4.02207	4.17E-09	-7.06156	7.52E-43	**IGFBP2**	NM_000597	-7.34569	5.5E-07	-2.65053	6.08E-05
**FAM117A**	NM_030802	-2.24983	9.39E-06	-2.72935	2.78E-19	**IGJ**	NM_144646	-4.1174	1.3E-07	-4.82787	2.59E-06
**FAM13A**	NM_001015045	-2.38213	1.72E-07	-2.01719	5.33E-08	**IL33**	NM_001199640	-2.70072	4.02E-05	-2.50953	5.1E-06
**FAM149A**	NM_001006655	-2.05322	0.00018	-3.11301	4.44E-13	**IPW**	NR_001293	-2.27418	0.000143	-3.52638	1.83E-15
**IRX3**	NM_024336	-7.71685	4.25E-11	-4.59295	4.45E-10	**MGAM**	NM_004668	-3.65687	1.81E-06	-3.11321	0.000207
**ITGA8**	NM_001291494	-2.27348	0.000111	-4.97686	3.79E-17	**MICALL1**	NM_033386	-2.13006	7.77E-07	-2.4273	3.27E-21
**ITM2A**	NM_001171581	-2.75877	7.05E-05	-3.06986	1.21E-14	**MRAP2**	NM_138409	-4.53033	4.91E-09	-2.34544	0.002534
**JAM2**	NM_001270407	-2.09847	9.26E-06	-4.25087	1.19E-24	**MT1E**	NM_175617	-2.85402	3.33E-07	-2.9049	5.81E-08
**KAT2B**	NM_003884	-2.05588	1.26E-06	-3.08241	6.26E-29	**MT1G**	NM_005950	-3.5684	9.5E-10	-3.29383	1.44E-05
**KCNE2**	NM_172201	-44.7496	1.12E-12	-8.63302	1.3E-11	**MT1M**	NM_176870	-14.1869	1.75E-11	-8.40764	8.9E-19
**KCNJ15**	NM_001276435	-8.79313	1.17E-08	-4.17348	3.27E-09	**MT1X**	NM_005952	-2.1321	4.66E-06	-3.41896	6.72E-21
**KCNJ16**	NM_001270422	-31.8379	1.35E-12	-6.30225	5.79E-08	**MT2A**	NM_005953	-2.02779	9.81E-07	-2.46336	1.72E-09

**KIT**	NM_000222	-4.99752	5.09E-11	-4.51202	2.65E-19	**MTTP**	NM_000253	-3.06604	0.043149	-10.7787	7.47E-12
**KLF15**	NM_014079	-2.09977	1.15E-05	-8.02801	1.57E-28	**MYH11**	NM_001040113	-2.87074	0.000394	-14.2578	2.94E-11
**KLF2**	NM_016270	-2.78586	5.67E-07	-2.82634	2.88E-11	**MYRIP**	NM_001284423	-10.3821	1.52E-09	-6.17162	4.56E-19
**KLF4**	NM_004235	-3.83427	9.16E-08	-3.58743	2.32E-18	**NEGR1**	NM_173808	-2.79674	2.52E-06	-5.38288	7.23E-24
**KLF9**	NM_001206	-2.33939	3.64E-06	-2.89866	2.31E-15	**NR3C1**	NM_000176	-2.1062	0.000232	-2.03177	7.72E-10
**KLK11**	NM_001136032	-9.85015	9.97E-09	-2.52663	0.049939	**NR3C2**	NM_000901	-2.84252	1.33E-05	-2.76411	5.73E-12
**KRT20**	NM_019010	-16.5946	6.36E-07	-5.93315	0.000292	**OGN**	NM_014057	-2.08347	0.04322	-5.79552	4.28E-11
**LAMA2**	NM_000426	-2.63415	1.04E-07	-2.15121	1.8E-05	**OTC**	NM_000531	-2.60634	0.011989	-5.6073	4.74E-05
**LEAP2**	NM_052971	-2.30216	0.001586	-2.13574	0.000571	**OXCT1**	NM_000436	-2.76985	1.48E-05	-2.11446	5.83E-07
**LEPR**	NM_001003679	-5.76493	3.49E-08	-2.02037	7.81E-08	**P2RY14**	NM_001081455	-2.61464	5.95E-05	-5.81074	4.95E-37
**LEPREL1**	NM_001134418	-3.02898	6.28E-06	-3.51828	9.7E-14	**PAIP2B**	NM_020459	-2.8792	3.53E-06	-4.32148	6.11E-18
**LIFR**	NM_001127671	-7.57047	1.51E-08	-3.37839	5.88E-12	**PBX1**	NM_001204961	-2.32623	0.00112	-2.59917	7.22E-11
**LIMCH1**	NM_001112717	-2.74479	6.7E-06	-2.00539	8.37E-08	**PDK4**	NM_002612	-3.81836	1.34E-05	-7.31146	1.07E-16
**LIPF**	NM_001198828	-107.27	1.31E-10	-34.4752	1.91E-12	**PDZD2**	NM_015022	-2.12794	2.49E-06	-2.35945	2.08E-11
**LOC400043**	NR_026656	-7.34448	7.33E-08	-2.57374	2.36E-05	**PER3**	NM_001289861	-3.46945	1.27E-11	-2.3882	2.44E-10
**LTF**	NM_001199149	-31.8009	1.25E-09	-4.17168	0.000522	**PGC**	NM_001166424	-38.4841	5.88E-08	-3.77718	0.026942
**MAL**	NM_002371	-13.6318	1.81E-14	-73.6867	1.12E-77	**PGM5**	NM_021965	-2.17237	0.000412	-12.2035	7.33E-27
**MAMDC2**	NM_153267	-4.00006	5.76E-08	-22.0044	5.79E-54	**PIK3C2G**	NM_001288772	-7.55981	1.6E-10	-4.19919	0.002435
**MAOA**	NM_000240	-2.40546	0.003705	-3.7122	6.22E-13	**PPP2R3A**	NM_001190447	-3.29698	2.78E-08	-2.87041	1.55E-15
**MAOB**	NM_000898	-2.47136	0.000217	-5.18117	2.48E-15	**PRIMA1**	NM_178004	-3.00878	6.85E-08	-18.7365	4.68E-41
**MAP7D2**	NM_001168465	-4.73573	5.58E-10	-6.0478	2.54E-08	**PRKAR2B**	NM_002736	-2.2776	0.000607	-3.82148	6.23E-21
**ME1**	NM_002395	-2.85637	3.22E-05	-2.73956	1.32E-12	**PSAPL1**	NM_001085382	-5.56235	6.55E-11	-8.16771	4.44E-07
**MEP1B**	NM_005925	-3.05428	0.001976	-7.59806	1.26E-08	**PSCA**	NM_005672	-27.5569	1.55E-08	-15.0785	1.27E-10
**METTL7A**	NM_014033	-5.89796	4.32E-09	-3.89504	1.07E-20	**PTGER3**	NM_000957	-4.35027	6.14E-06	-3.31149	6.65E-10
**MFAP4**	NM_001198695	-2.36524	0.003092	-4.89536	3.35E-12	**PTGR1**	NM_001146108	-2.54201	6.09E-06	-3.42398	7.74E-22
**MFAP5**	NM_003480	-3.26845	0.000181	-3.57639	1.72E-09	**PTGS1**	NM_000962	-2.04548	1.12E-07	-4.28324	2.05E-26

**MFSD4**	NM_181644	-37.1663	5.38E-12	-2.4867	0.001262	**PTPRS**	NM_002850	-2.4842	4.42E-09	-3.03852	2.39E-10
**PTPRZ1**	NM_001206838	-7.0904	2.07E-07	-7.64456	5.86E-17	**SOX2**	NM_003106	-3.51156	5.16E-06	-5.58148	1.22E-09
**RAB30**	NM_001286059	-2.56892	2.77E-07	-2.75484	2.04E-18	**SPAG16**	NM_001025436	-4.79659	8.55E-07	-3.02148	2.57E-13
**RAI2**	NM_001172732	-2.67528	2.07E-06	-3.06995	1.9E-14	**SRPX**	NM_001170750	-3.29552	1.33E-05	-4.7241	1.97E-23
**RBP2**	NM_004164	-3.09277	0.018144	-12.2826	7.13E-09	**SST**	NM_001048	-22.2206	2.71E-11	-20.2492	9.7E-16
**RCAN2**	NM_001251973	-3.28312	8.46E-08	-3.58299	7.32E-19	**SYNPO2**	NM_001128933	-3.24423	0.000372	-7.87079	3.35E-10
**RDH12**	NM_152443	-6.9514	6.83E-10	-13.5532	2.55E-29	**SYTL5**	NM_001163334	-10.3631	5.04E-12	-2.76633	0.00332
**REG3A**	NM_002580	-25.6101	2.27E-08	-8.97833	0.00013	**TCEA3**	NM_003196	-2.71173	0.00035	-2.4568	3E-06
**RGMB**	NM_001012761	-3.65941	8.56E-08	-2.59025	1.09E-14	**TCEAL3**	NM_001006933	-2.20058	8.42E-05	-2.23694	1.38E-12
**RGN**	NM_001282848	-2.092	0.002106	-5.4689	6.79E-14	**TEF**	NM_001145398	-2.02164	1.43E-08	-2.76956	1.27E-24
**RNASE1**	NM_002933	-5.15942	1.22E-09	-3.09751	1.41E-10	**TFF2**	NM_005423	-9.06992	1.03E-06	-4.47006	0.00663
**ROR1**	NM_001083592	-2.51681	8.6E-05	-2.18915	9.26E-09	**TLE4**	NM_001282748	-2.2072	6.49E-06	-2.45557	3.56E-17
**RORA**	NM_002943	-2.28235	0.000374	-2.13694	2.58E-11	**TLR3**	NM_003265	-2.33818	0.000925	-2.62947	1.75E-11
**SCARA5**	NM_173833	-5.56913	1.39E-09	-23.3207	3.56E-54	**TMEM100**	NM_001099640	-6.55994	2.73E-11	-12.3918	3.74E-40
**SCGB2A1**	NM_002407	-16.7182	9.11E-13	-7.50493	2.4E-07	**TMEM220**	NM_001004313	-4.52038	1.2E-11	-3.23653	3.28E-20
**SCIN**	NM_001112706	-6.49708	3.48E-07	-3.85196	5.16E-09	**TMEM27**	NM_020665	-3.27391	8.18E-09	-2.13332	1.35E-05
**SCNN1G**	NM_001039	-5.24863	9.75E-07	-23.3364	1.96E-27	**TMEM37**	NM_183240	-2.52746	3.13E-07	-2.18497	5.46E-06
**SDPR**	NM_004657	-2.75956	2.58E-05	-3.86266	3.02E-17	**TMPRSS15**	NM_002772	-4.68941	0.007099	-14.7849	1.31E-11
**SELENBP1**	NM_001258288	-4.16363	5.3E-07	-2.5674	1.12E-09	**TNFRSF17**	NM_001192	-10.1114	9.31E-10	-2.68933	0.006576
**SETBP1**	NM_001130110	-2.29056	1.27E-06	-3.07632	6E-13	**TOX**	NM_014729	-2.79659	4.66E-05	-3.07574	8.64E-11
**SFRP1**	NM_003012	-2.14131	0.001461	-15.8511	8.61E-35	**TPD52L1**	NM_001003395	-3.16588	0.000259	-3.42094	9.36E-09
**SGK1**	NM_001143676	-2.60043	9.78E-11	-2.05527	9.67E-09	**TPH1**	NM_004179	-5.48383	4.52E-08	-2.92853	0.00046
**SIDT2**	NM_001040455	-2.76201	5.93E-11	-2.01881	1.2E-16	**TPSAB1**	NM_003294	-3.18486	2.19E-05	-3.5427	3.52E-09
**SLAIN1**	NM_001040153	-3.26485	2.08E-05	-2.05507	0.000143	**TPSB2**	NM_024164	-2.8879	8.01E-05	-3.60521	2.89E-09
**SLC16A7**	NM_001270622	-6.67104	1.4E-08	-3.62867	2.18E-12	**TSC22D3**	NM_001015881	-2.75225	2.12E-05	-3.54073	7.33E-19
**SLC25A4**	NM_001151	-2.33397	2.03E-06	-3.68267	4.22E-25	**UBE2QL1**	NM_001145161	-4.21417	1.18E-07	-5.5726	1.35E-23

**SLC26A7**	NM_001282356	-7.72837	3.07E-08	-2.00072	0.014573	**UPK1B**	NM_006952	-2.99533	0.004618	-6.28298	2E-05
**SLC28A2**	NM_004212	-2.62773	0.017131	-5.97838	0.000233	**VLDLR**	NM_001018056	-2.12708	1.41E-05	-3.43796	2.1E-13
**SLC2A12**	NM_145176	-4.47873	6.17E-07	-3.17161	3.06E-15	**VSIG2**	NM_014312	-15.4196	8.95E-11	-3.57679	0.001298
**SLC7A8**	NM_001267036	-3.99142	2.18E-11	-3.26641	2.84E-16	**WFDC1**	NM_001282466	-2.4006	0.000178	-2.86406	5.16E-13
**SMAD9**	NM_001127217	-4.16132	8.6E-08	-3.48219	3.81E-12	**WIPF3**	NM_001080529	-3.31417	7.97E-07	-2.41684	9.96E-05
**SNRPN**	NM_003097	-2.57375	3.94E-06	-3.8849	2.59E-23	**XK**	NM_021083	-3.17431	1.64E-05	-2.05408	0.000281
**SOBP**	NM_018013	-2.26129	2.53E-05	-2.78007	8.17E-13	**ZBTB7C**	NM_001039360	-3.94262	1.96E-09	-4.92124	8.4E-12
**SORBS1**	NM_001034954	-3.25029	9.24E-05	-4.51225	5.94E-11	**ZG16**	NM_152338	-2.71276	0.006832	-7.43114	2.73E-08
**SORBS2**	NM_001145670	-3.0045	7.34E-09	-3.90647	5.41E-15						
**SOSTDC1**	NM_015464	-32.5967	5.75E-12	-23.8444	4.59E-41						

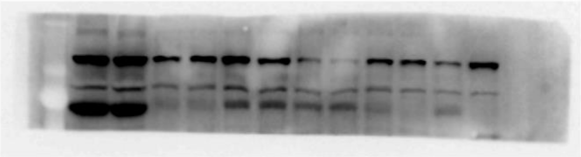

Sup-figure 1b. Calpain-8

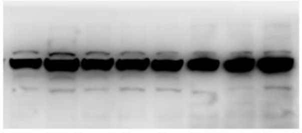

Sup-figure 1d. Calpain-8

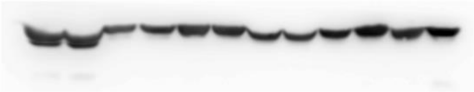

Sup-figure 1b. β-Actin

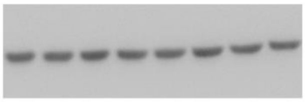

Sup-figure 1d. β-Actin

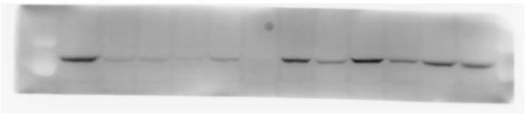

Sup-figure 1b. Calpain-9

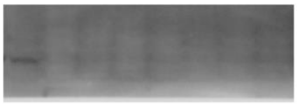

Sup-figure 1d. Calpain-9

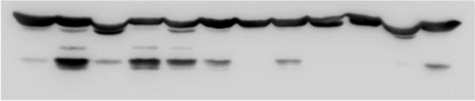

Sup-figure 1b. β-Actin

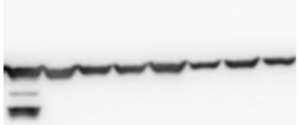

Sup-figure 1d. β-Actin

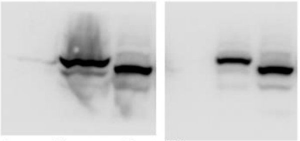

Sup-figure 2a. Flag

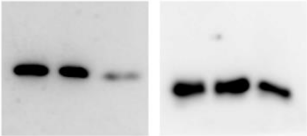

Sup-figure 2d. CDK4

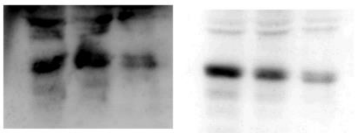

Sup-figure 2d. Cyclin D1

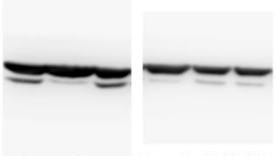

Sup-figure 2a. GAPDH

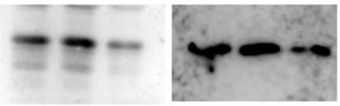

Sup-figure 2d.CDK6

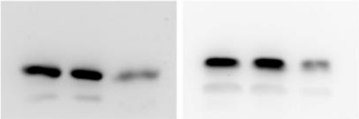

Sup-figure 2d. Cyclin D3

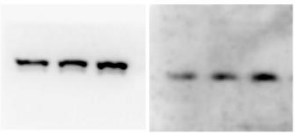

Sup-figure 2d. p21

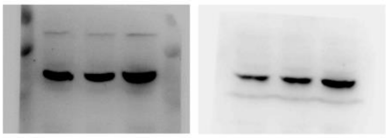

Sup-figure 2f. cleaved &Caspase12

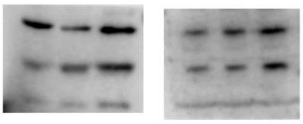

Sup-figure 2f. Cleaved Caspase9

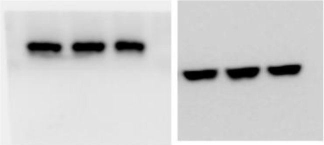

Sup-figure 2d. GAPDH

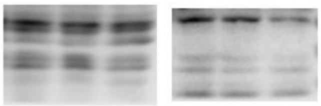

Sup-figure 2f. Cleaved Caspase8

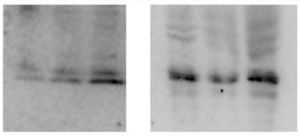

Sup-figure 2f. Cleaved Caspase3

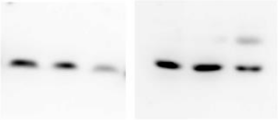

Sup-figure 2f. Bcl-2

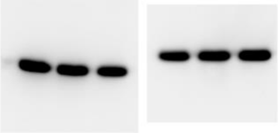

Sup-figure 2f.GAPDH

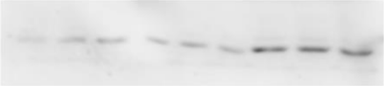

Sup-figure 3c. Cleaved Caspase12

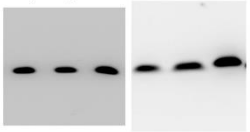

Sup-figure 2f. Bax

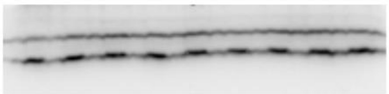

Sup-figure 3c. B-actin
